# Sequential co-reduction of nitrate and carbon dioxide enables selective urea electrosynthesis

**DOI:** 10.1038/s41467-023-44131-z

**Published:** 2024-01-02

**Authors:** Yang Li, Shisheng Zheng, Hao Liu, Qi Xiong, Haocong Yi, Haibin Yang, Zongwei Mei, Qinghe Zhao, Zu-Wei Yin, Ming Huang, Yuan Lin, Weihong Lai, Shi-Xue Dou, Feng Pan, Shunning Li

**Affiliations:** 1 School of Advanced Materials, Peking University, Shenzhen Graduate School, Shenzhen, Guangdong 518055 China; 2 Hydrogen Energy Institute, Zhejiang University, Hangzhou, Zhejiang 310027 China; 3 Institute of Fundamental and Frontier Sciences, University of Electronic Science and Technology of China, Chengdu, 611731 China; 4 Institute of Chemistry, Chinese Academy of Sciences, Beijing, 100190 China; 5 Institute for Superconducting and Electronic Materials, University of Wollongong, Wollongong, NSW 2522 Australia

**Keywords:** Catalysis, Electrochemistry

## Abstract

Despite the recent achievements in urea electrosynthesis from co-reduction of nitrogen wastes (such as NO_3_^−^) and CO_2_, the product selectivity remains fairly mediocre due to the competing nature of the two parallel reduction reactions. Here we report a catalyst design that affords high selectivity to urea by sequentially reducing NO_3_^−^ and CO_2_ at a dynamic catalytic centre, which not only alleviates the competition issue but also facilitates C−N coupling. We exemplify this strategy on a nitrogen-doped carbon catalyst, where a spontaneous switch between NO_3_^−^ and CO_2_ reduction paths is enabled by reversible hydrogenation on the nitrogen functional groups. A high urea yield rate of 596.1 µg mg^−1^ h^−1^ with a promising Faradaic efficiency of 62% is obtained. These findings, rationalized by in situ spectroscopic techniques and theoretical calculations, are rooted in the proton-involved dynamic catalyst evolution that mitigates overwhelming reduction of reactants and thereby minimizes the formation of side products.

## Abstract (web summary)

Electrochemical co-reduction of nitrate and carbon dioxide is a promising approach for urea synthesis. Here, the authors report a sequential co-reduction strategy for catalyst design that can dynamically adjust the competition between the two reduction processes, resulting in a high selectivity towards urea.

## Introduction

The recent surge in literature devoted to electrochemical synthesis of urea has been fuelled by the desperate need for both energy conservation and CO_2_ fixation to mitigate climate change. As one of the most frequently used nitrogen fertilizers^1,2^, urea can be synthesized at the industrial level via the consecutive reactions of N_2_ + H_2_ → NH_3_ and NH_3_ + CO_2_ → urea, both of which are energy-intensive and require harsh conditions^3–8^. Renewable electricity-driven production of urea using nitrogen species (e.g., N_2_, nitrate, nitrite, NO) and CO_2_ as feedstocks^9–21^, offers a promising alternative to the conventional route (Supplementary Fig. 1), but there is still a lack of studies that embody practical solutions to urea electrosynthesis at large scale^22,23^. The most daunting challenge is to discover a selective catalyst for efficient C−N coupling after moderate hydrogenation of the reactants, while inhibiting their conversion into side products such as NH_3_ and CO. In regard to hydrogenation and C−N coupling process, the fixed nitrogen is more appealing than N_2_ as the nitrogen source, since N_2_ molecule has an exceedingly high dissociation energy for the triple bond (941 kJ mol^−1^) and an inferior solubility in water (6.24 × 10^−4^ mol L^−1^ atm^−1^)^24–26^. Although direct reaction between adsorbed N_2_ and CO was proposed, kinetic restrictions would prevent the attainment of a satisfactory yield rate of urea. In contrast, electrochemical co-reduction of nitrate (NO_3_^−^) and CO_2_ is much easier to realize^10,13,14^, which, when steered towards urea formation, has the tantalizing prospect of industrialization given the easy access of NO_3_^−^ from industrial wastewater and domestic sewage (Supplementary Fig. 2)^27–29^. The problem, however, is that the hydrogenation processes of NO_3_^−^ and CO_2_ actually compete with each other, not to mention that both processes should take place at virtually the same site to permit facile C−N coupling^20,21,30^. Indeed, with few exceptions, side reactions always predominate over urea formation on a variety of electrocatalysts due to the overwhelming reduction of one reactant over the other^25,31–35^.

**Fig. 1:**
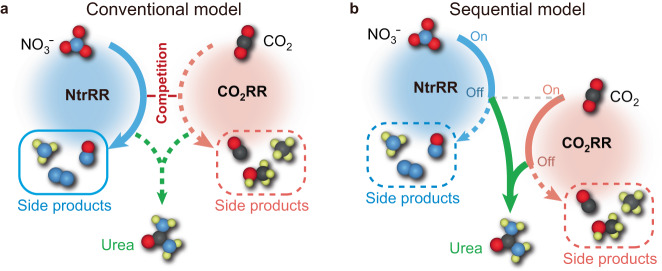
Conventional and sequential models for urea electrosynthesis. **a** Concurrent occurrence of NtrRR and CO_2_RR, leading to inferior production of urea and pronounced formation of side products, such as those from NtrRR. **b** Sequential combination of NtrRR and CO_2_RR, successively biasing the competition between both reactions in favour of one and resulting in high selectivity for urea synthesis. Colour codes: N, blue; O, red; C, black; H, yellow.

To alleviate the competition between concurrent NO_3_^−^ and CO_2_ reduction, time-staggering of both reactions could serve as a viable strategy; that is, the reduction of each reactant is spatially coincident but temporally separated and favoured at different stages. Figure 1a depicts the reaction timeline of the conventional mechanism in the reduction process for urea synthesis, with NO_3_^−^ expected to be relatively more reactive than CO_2_ on the catalyst. In comparison, the sequential mechanism (Fig. 1b) avoids the predominance of nitrate reduction reaction (NtrRR) along the whole reaction path. In the beginning, NtrRR is assumed to take place prior to CO_2_ reduction reaction (CO_2_RR), and at this stage, CO_2_RR is suspended due to inferior kinetics. At the point where NtrRR process encounters a major reaction barrier, the further reduction of the corresponding intermediate is halted, with CO_2_RR simultaneously switched on. Before the release of CO_2_RR products, C−N coupling utilizing the preformed NtrRR intermediates nearby should take place and direct the reaction to urea formation. As a prerequisite for this sequential reduction process, the catalyst should be capable of switching its catalytic activity in favour of CO_2_RR after the initiation of NtrRR. However, we know of no previous attempts to explore such catalyst. Thanks to recent studies reporting the Faradaic pseudocapacitance behaviour of nitrogen-doped carbon (NC) materials^36,37^, we were intrigued by the fact that the nitrogen species in *sp*^2^-hybridized carbon could undergo reversible hydrogenation, implying their possible use as dynamic active sites during catalysis. The feasibility of reversible formation and cleavage of N−H bonds on NC could provide an attractive platform for self-tuning of the catalytic activity^38,39^, and we speculate that this feature could regulate the formation sequence of intermediates during co-reduction reactions.

In this work, we compare the electrochemical performance between a defective NC catalyst and a Cu_1_/NC single-atom catalyst that is prepared using the same procedure as the former except for the addition of a Cu precursor. Previous studies have revealed that the activity of pyridinic/pyrrolic nitrogen-coordinated single-atom catalysts is mainly derived from the isolated metal centres^40,41^. In particular, single-atom Cu species were perceived to be active for catalysing NtrRR^25^ and CO_2_RR^42^. Here we demonstrate that while Cu_1_/NC excels in NtrRR along with decent activity for CO_2_RR, the strong competition between both reduction processes throughout the whole reaction path results in low production of urea. In contrast, the NC catalyst not only triggers sequential reduction of NO_3_^−^ and CO_2_, but also enables facile C−N coupling, which confers extraordinary catalytic performance for urea electrosynthesis. Benefiting from these features, a urea yield rate of 596.1 μg mg^−1^ h^−1^ with a Faradaic efficiency (FE) of 62% is achieved on NC at −0.5 V versus reversible hydrogen electrode (RHE), which is superior to most of the previously reported catalysts. This sequential reduction behaviour stems from the reaction-driven evolution of the NC catalyst, shaping a seesaw scenario: the seesaw of the reaction is initially tilted to NtrRR in the presence of C=N−H species on the catalyst, during which the N−H bonds are cleaved and the catalytic centres become activated for CO_2_RR, thus tipping the seesaw over and turning the reaction to *CO formation and further C−N coupling. After the whole reaction process, the catalytic centres would be spontaneously restored to the initial state. This dynamic reversibility can endow a high propensity for urea formation and hence gives rise to the unprecedented inhibition of side reactions, which offers a strategy to design highly selective catalysts for urea electrosynthesis.

## Results

### Structural characterization

**Fig. 2:**
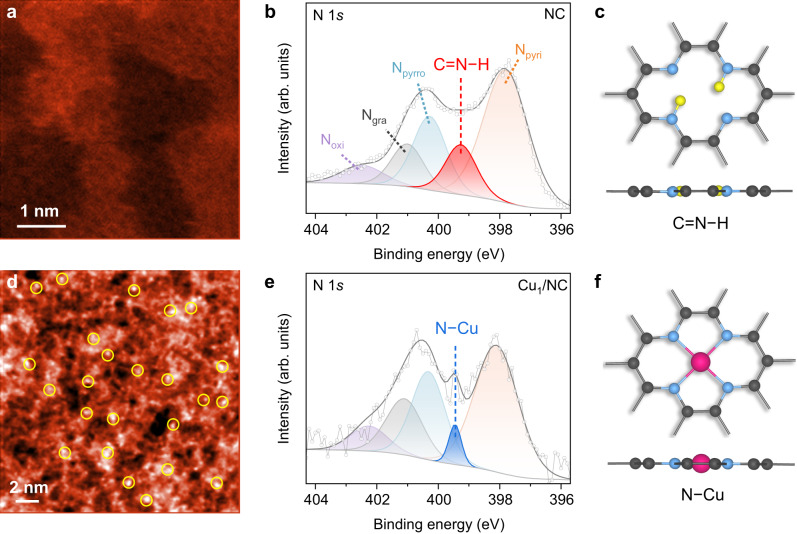
Structural characterization of NC and Cu_1_/NC. **a**, **d** HAADF-STEM images of NC (**a**) and Cu_1_/NC (**d**). **b**, **e** N 1*s* XPS spectra of NC (**b**) and Cu_1_/NC (**e**). **c**, **f** Local structures of typical C=N−H species in NC (**c**) and N−Cu species in Cu_1_/NC (**f**). Colour code: N, blue; C, black; H, yellow; Cu, pink.

The NC was prepared by a simple one-pot pyrolysis method using glucose and dicyandiamide as carbon and nitrogen sources, respectively^9,43–45^, while Cu_1_/NC was obtained by further addition of Cu(NO_3_)_2_ (Supplementary Fig. 3). X-ray diffraction (XRD) result of NC shows a broad diffraction peak at 21.1° (Supplementary Fig. 4), which can be attributed to the typical graphitic (002) plane. An identical pattern is displayed for Cu_1_/NC, implying the absence of metal nanoparticles. No peak can be assigned to the in-plane long-range order structure of C_3_N_4_ for both samples. Electron microscopy results substantiate the sheet-like morphology of both samples and reveal an abundance of folds and wrinkles (Supplementary Figs. 5–7). Figure 2a, d presents the aberration-corrected high-angle annular dark-field scanning transmission electron microscopy (HAADF-STEM) images of NC and Cu_1_/NC, with the latter exhibiting bright spots that correspond to single Cu atoms dispersed across the substrate. Energy dispersive X-ray spectroscopy (EDS) elemental mapping indicates a homogenous distribution of nitrogen in the NC sample (Supplementary Fig. 6). This homogenous distribution is well preserved in Cu_1_/NC (Supplementary Fig. 7), where the nitrogen species could serve as effective sites for capturing and dispersing the Cu atoms. The atomic dispersion of Cu is confirmed by extended X-ray absorption fine structure (EXAFS) spectroscopy, showing the presence of a strong peak assigned to the Cu-N bond (Supplementary Fig. 8). Fourier-transform infrared (FTIR) spectroscopy further indicates the formation of N−H species in the π-conjugated network of graphitic carbon (Supplementary Fig. 9)^46^. Electrons donated by the N atoms can be directly probed by the electron paramagnetic resonance (EPR) spectra (Supplementary Fig. 10). Moreover, the co-appearance of *sp*^2^ and *sp*^3^ peaks in the Raman spectrum of NC (Supplementary Fig. 11) indicates the existence of numerous defects in the architecture, which are inherent to the calcination process during synthesis. Related to this feature is a high specific surface area of 879.59 m^2^ g^−1^ as estimated from the Brunauer–Emmett–Teller (BET) plots, and a similar value holds for Cu_1_/NC (Supplementary Figs. 12 and 13). We note that no obvious trace of C_3_N_4_ has been detected in all the FTIR and Raman spectra.

X-ray photoemission spectroscopy (XPS) was carried out to characterize the nitrogen and Cu species on NC and Cu_1_/NC (Supplementary Figs. 14–16). The N 1*s* spectrum of NC (Fig. 2b) can be deconvoluted into components corresponding to pyridinic N (N_pyri_, 397.9 eV), pyrrolic N (N_pyrro_, 400.3 eV), graphitic N (N_gra_, 401.0 eV), oxidized N (N_oxi_, 402.5 eV) and C=N−H (399.3 eV)^47–50^. For the N 1*s* XPS spectrum of Cu_1_/NC (Fig. 2e), a sharp peak emerges at 399.5 eV, which can be indexed to the N−Cu bonds^9,51,52^, replacing the broad C=N−H peak. Accordingly, we may expect that the incorporation of Cu single atoms can suppress the N−H bonds at the pyridinic/pyrrolic N atoms in NC. These results coincide with the recent work^49^ that demonstrated the dehydrogenation of nitrogen-doped carbon at the anchoring sites of transition-metal atoms during the synthesis of single-atom catalysts. We have performed density functional theory (DFT) calculations to assess the energetics of N−H bond formation on NC (Supplementary Fig. 17). A configuration of four neighbouring pyridinic N (denoted as N_4_) in a graphene sheet, which is identical to the Cu-N_4_ moiety^53^ in Cu_1_/NC (Fig. 2f) but without the presence of Cu, was taken as the model system for the study of C=N−H species. The binding of the first H atom to one of the N atoms is highly exothermic, with a free energy change (∆*E*_1H_) of −0.72 eV at 0 V versus RHE. The second H preferentially adsorbs onto another N atom furthest from the former one, giving a free energy change (∆*E*_2H_) of −0.62 eV. Since the successive binding of the next two H atoms to the remaining N atoms will consume substantial energy (1.16 and 1.23 eV, respectively), they are unlikely to be trapped at experimental conditions. Consequently, each pyridinic N_4_ moiety will spontaneously capture up to two H atoms (Fig. 2c). Their reversible removal in electrochemical reactions has constituted the premise of Faradaic pseudocapacitance for NC^36,37^.

### Electrocatalytic performance for urea synthesis

**Fig. 3:**
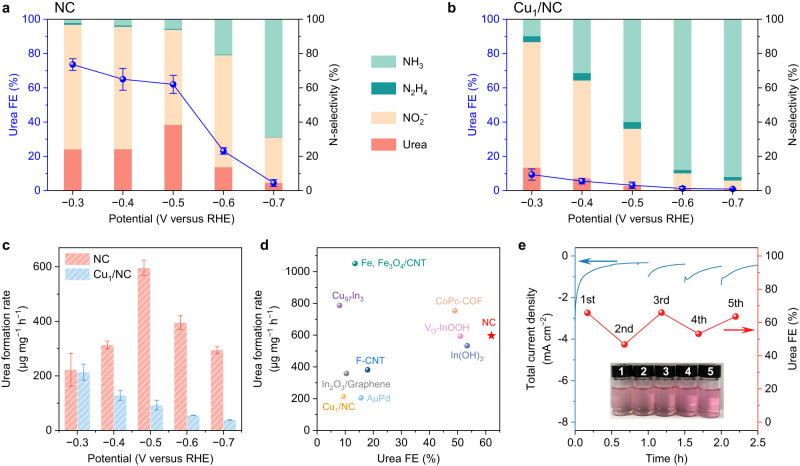
Electrocatalytic performance of urea synthesis on NC and Cu_1_/NC. **a**, **b** The FEs of urea and the N-selectivity of all N-containing products under different potentials on NC (**a**) and Cu_1_/NC (**b**). **c** Urea yield rates at different potentials. **d** Comparison of urea formation rate and FE between NC and other catalysts reported in the literatures, including Fe + Fe_3_O_4_ at carbon nanotubes (Fe, Fe_3_O_4_/CNT)^71^, In(OH)_3_^14^, oxygen-deficient InOOH (V_O_-InOOH)^72^, F-doped carbon nanotubes (F-CNT)^73^, Cu_97_In_3_^74^, phthalocyanine-based covalent organic framework (CoPc-COF)^75^, In_2_O_3_/Graphene^76^, and AuPd^77^. Corresponding values are tabulated in Supplementary Table 1. **e** Stability test of NC at −0.5 V versus RHE for 5 cycles. The inset shows that the colour of the solution reacted with diacetyl monoxime is almost identical during cycling.

The evaluation of electrocatalytic selectivity of NC and Cu_1_/NC for urea synthesis was performed in an H-type cell (Supplementary Fig. 18) via the chrono-amperometry (CA) method. An electrolyte composed of 0.1 M KHCO_3_ and 0.1 M KNO_3_ was adopted, and high-purity CO_2_ was continuously bubbled to maintain saturation during the electrolysis. The concentration of urea was measured by diacetyl monoxime method (Supplementary Fig. 19)^14^. Besides urea, a series of side products, including ammonia (NH_3_), nitrite (NO_2_^−^), hydrazine (N_2_H_4_), carbon monoxide (CO) and hydrogen (H_2_), were also identified through spectrophotometric and gas chromatographic analysis (Supplementary Figs. 20–22). The product yields at each potential were averaged over three independent measurements. As displayed in Fig. 3a and Supplementary Fig. 24, urea is the predominant product on NC catalyst from −0.3 to −0.5 V versus RHE. According to the molar yields of N-containing products, the N-selectivity was obtained, showing a relatively high proportion of NO_3_^−^ converted into NO_2_^−^. In spite of this, the FE of NO_2_^−^ product is rather limited as compared with that of urea, given the much smaller number of transferred electrons for conversion into NO_2_^−^ than urea. Moreover, the hydrogen evolution reaction (HER) is significantly suppressed on NC (Supplementary Fig. 25) as compared to those previously designed catalysts for urea electrosynthesis^10^. This feature represents one of the most critical attributes of the superior urea selectivity on NC.

In contrast, Cu_1_/NC presents an FE of below 15% towards urea with excessive formation of side products (Fig. 3b), among which NtrRR products (NO_2_^−^ and NH_3_) take the lead in the whole potential range investigated. Given the nearly identical synthesis procedure for both NC and Cu_1_/NC, their major difference likely stems from the C=N−H and N−Cu species based on whether Cu ions were incorporated. This speculation was substantiated by the XPS results shown in Fig. 2b, e. Thus, we may postulate that the huge difference in the performance of urea synthesis should be attributed to the above two species, both of which could serve as the catalytic centres for the reduction of NO_3_^−^ and CO_2_. Moreover, we have examined other single-atom catalysts, including In_1_/NC and Fe_1_/NC, to co-reduce NO_3_^−^ and CO_2_ (Supplementary Fig. 26). Only negligible amounts of urea are formed for both catalysts due to the competitive NtrRR. The seesaw between NO_3_^−^ and CO_2_ reduction is always tilted to the former, thus hindering C−N coupling, which appears to be a common situation in carbon-supported single-atom catalysts.

The effect of applied potentials on the yield rate of urea is displayed in Fig. 3c. As compared with Cu_1_/NC, the NC catalyst produces considerably higher yields at −0.5 V versus RHE. Despite a larger total current density delivered by Cu_1_/NC (Supplementary Fig. 27), it consumes most electrons to generate side products, resulting in remarkably poor selectivity to C−N coupling. C−N coupling is a potential-independent step, and when the bias potential is sufficiently negative, this reaction step would be kinetically less favourable than the excessive reduction of the intermediates into side products. This can rationalize the decreasing urea yields for NC at −0.5 to −0.7 V versus RHE. Notably, the NC catalyst enables urea electrosynthesis at a maximum yield rate of 596.1 μg mg^−1^ h^−1^ with a promising FE of 62% under −0.5 versus RHE, which is superior to most of the recently reported catalysts working at similar potentials (Fig. 3d). The durability test of NC showed almost no degradation in either activity or urea selectivity for 5 successive runs (Fig. 3e and Supplementary Fig. 28). TEM measurements were further performed after the test (Supplementary Fig. 29), showing that the morphology of NC catalyst remains largely intact. The sustained electrocatalytic activity not only demonstrates the long-term stability of NC, but also indicates that the nitrogen and carbon sources originate from NO_3_^−^ and CO_2_ rather than from the pyridinic/pyrrolic N and carbon atoms in the catalyst. In addition, we have synthesized another NC sample at an elevated pyrolysis temperature, which results in a greatly reduced amount of C=N−H species and exhibits a much lower urea FE in the co-reduction reaction (Supplementary Figs. 30 and 31). This implies that C=N−H plays a pivotal role for urea synthesis on NC.

### Control experiments for mechanistic rationalization

**Fig. 4:**
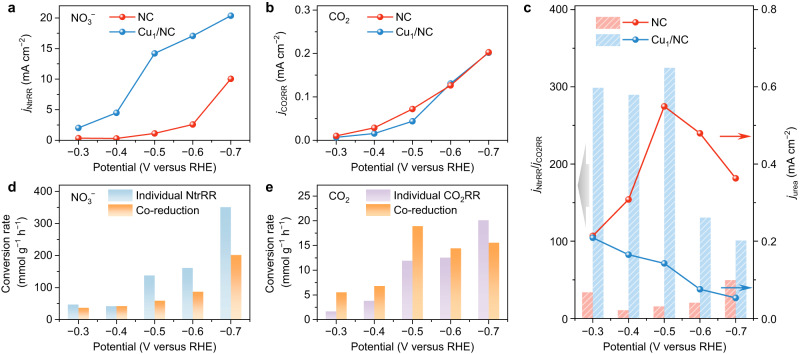
Control experiments of individual NtrRR and individual CO_2_RR on NC and Cu_1_/NC. **a** NtrRR current density (*j*_NtrRR_, corresponding to the sum of partial current densities of NO_2_^−^, N_2_H_4_ and NH_3_) during the individual reduction of NO_3_^−^. **b** CO_2_RR current density (*j*_CO2RR_, corresponding to the partial current density of CO) during the individual reduction of CO_2_. **c** The ratio between *j*_NtrRR_ and *j*_CO2RR_, and the urea partial current density (*j*_urea_) during the co-reduction of NO_3_^−^ and CO_2_. **d** The conversion rate of NO_3_^−^ to N-containing products on NC in individual NtrRR as compared with co-reduction reaction. **e** The conversion rate of CO_2_ to C-containing products on NC in individual CO_2_RR as compared with co-reduction reaction.

To better understand the electrocatalytic property of NC and Cu_1_/NC, we performed control experiments to evaluate their activity for NtrRR and CO_2_RR separately. In the electrochemical tests of individual NtrRR, argon gas instead of CO_2_ was fed into the electrolyte. Over the NC catalyst, NO_2_^−^ was detected to be the major product at −0.3~−0.5 V versus RHE (Supplementary Fig. 34), while NH_3_ formation predominated on Cu_1_/NC with FE reaching nearly 100% at negative potentials exceeding −0.5 V versus RHE. We find that Cu_1_/NC can deliver a much higher NtrRR current density (including the formation of NO_2_^−^, N_2_H_4_ and NH_3_) than NC (Fig. 4a). Given that NH_3_ is the most reduced product along the reaction path of NtrRR, the above results suggest that the replacement of C=N−H by N−Cu could promote the maximum reduction of NO_3_^−^. Intuitively, N−Cu may seem beneficial for urea electrosynthesis because the formation of urea (CO(NH_2_)_2_) also requires the maximum reduction of N in NO_3_^−^ to a valency of −3. However, as shown below, the urea selectivity would actually be adversely impacted by the facile NtrRR process when the CO_2_RR process shows much inferior activity to NtrRR. Figure 4b and Supplementary Fig. 35 present the results of individual CO_2_ reduction experiments, in which NO_3_^−^ was removed from the electrolyte and the catalysts were run under a CO_2_ gas flow. While the derived CO_2_RR current densities are similar between NC and Cu_1_/NC, they are one order of magnitude smaller than the NtrRR current densities in individual NO_3_^−^ reduction. DFT calculation results corroborate this finding, revealing that the first hydrogenation step of CO_2_, i.e., *CO_2_ → *COOH, is rate-determining and strongly endothermic (>1.3 eV) for both NC and Cu_1_/NC (Supplementary Fig. 36). With the balance between NtrRR and CO_2_RR struck during co-reduction of NO_3_^−^ and CO_2_, there would be limited opportunity for urea formation at a single catalytic centre if the activity of this site remains unchanged in the reactions.

Cu_1_/NC conforms to the above scenario, which is best seen when the potential becomes more negative. On Cu_1_/NC, activity for both individual NtrRR and individual CO_2_RR are obviously promoted from −0.3 to −0.7 V versus RHE (Fig. 4a, b), whereas the yield of urea shows a descending trend (Fig. 3c). A fiercer competition between both reactions at more negative potentials engages the co-reduction process into a more imbalanced state. The predominating NtrRR process, which takes more advantages of the bias potential than the potential-independent C−N coupling process, would thereby significantly inhibit urea formation. Unlike Cu_1_/NC, the NC catalyst can steer the co-reduction of NO_3_^−^ and CO_2_ along the urea formation path. We show in Fig. 4c that as compared to Cu_1_/NC, the ratio between NtrRR and CO_2_RR current densities in the control experiments is considerably smaller. Obviously, NtrRR on NC occupies a less predominating position than that on Cu_1_/NC, which could offer more chance for C−N coupling during the co-reduction reaction. Another point worth mentioning is that on NC, the conversion rate of CO_2_ as obtained from the yield rates of urea and CO (in mmol g^−1^ h^−1^) in co-reduction surpasses that in individual CO_2_RR from −0.3 to −0.6 V versus RHE (Fig. 4e). This clearly suggests that the electrochemical conversion of CO_2_ could be activated in the presence of NO_3_^−^. According to DFT calculations, we can rule out the possibility of C−N coupling between CO_2_ and the main intermediates of NtrRR on NC (Supplementary Fig. 37). Hence, the hydrogenation of CO_2_, especially the rate-determining *CO_2_ → *COOH step in CO_2_RR, is required for urea formation. In this context, the promoted CO_2_ conversion would imply that the NtrRR process plays a nontrivial role in reducing energy consumption at this elementary step. Such reduction can be interpreted as an NtrRR-induced alteration of the catalyst activity.

Notably, we find that on NC catalyst, the conversion rate of NO_3_^−^ is either minimally affected or considerably reduced upon the introduction of CO_2_ as a reactant (Fig. 4d). It means that the NtrRR intermediates can participate in the urea formation process but seems not able to be activated from the CO_2_RR intermediates. NtrRR is even hindered by the presence of CO_2_ to some extent. Considering that the onset potential of NtrRR is more positive than that of CO_2_RR (Supplementary Fig. 38), it can be safely inferred that NtrRR precedes CO_2_RR during urea synthesis. In this context, NO_3_^−^ is first reduced at the catalytic centre and the reaction proceeds to NO_2_^−^ via two hydrogenation steps (valency of N changing from +5 to +3). Since the FE of NO_2_^−^ in individual NtrRR is nearly 100% at −0.3~−0.4 V versus RHE (Supplementary Fig. 34), only a negligible amount of NO_2_^−^ can be further reduced in this potential range, which can eliminate the possibility that the reduction products beyond NO_2_^−^ could participate in C−N coupling during co-reduction. This suggests that the switch of reaction path to CO_2_RR is most likely accomplished in the period of forming NO_2_^−^. Subsequently, CO_2_RR occurs in the vicinity of the previously formed NtrRR intermediates/products, and the CO_2_RR intermediates can readily combine with these N-containing species to form urea. The above considerations have prompted us to propose that the co-reduction of NO_3_^−^ and CO_2_ on NC adopts a sequential model via a consecutive switch between two reduction reactions.

### Operando tracking of the surface species

**Fig. 5:**
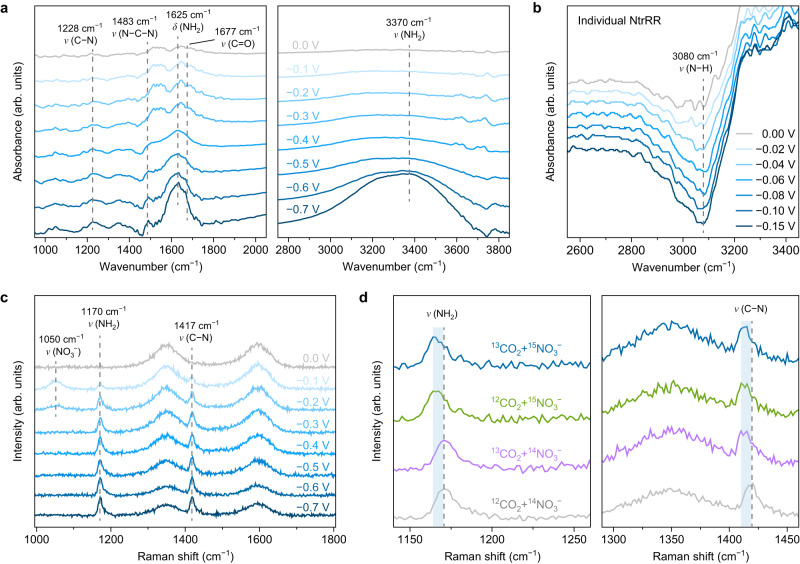
Operando attenuated total reflection surface-enhanced infrared absorption spectroscopy (ATR-SEIRAS) and isotope-labelled in situ Raman characterization. **a** ATR-SEIRAS spectra for NC under different applied potentials during co-reduction of CO_2_ and NO_3_^−^. **b** ATR-SEIRAS spectra for NC in the range of relatively low applied potentials during individual NtrRR. **c** In situ Raman spectra for NC under different applied potentials during co-reduction of CO_2_ and NO_3_^−^. **d** Comparison of the in situ Raman spectra under isotope-labelled NO_3_^−^ and CO_2_ at –0.50 V versus RHE.

Operando attenuated total reflection surface-enhanced infrared absorption spectroscopy (ATR-SEIRAS) can provide critical details to better understand the NO_3_^−^ and CO_2_ co-reduction mechanism on NC catalyst. Figure 5a shows the ATR-SEIRAS spectra with applied potentials varying from 0 to −0.7 V versus RHE, where several infrared bands are detected in the wavenumber ranges of 1000–2000 and 2800–3800 cm^−1^. Two bands at around 1625 and 3370 cm^−1^ could be assigned to the bending and stretching (including symmetrical and antisymmetrical components) modes of −NH_2_, respectively^14,54^, both of which show a marked increase in intensity from −0.5 to −0.7 V. This is consistent with the surge in production of NH_3_ at voltages approaching −0.7 V as revealed in electrochemical experiments. The bands centred at around 1483 and 1288 cm^−1^ correspond to the antisymmetrical N−C−N stretching vibration of urea and the C−N stretching vibration of the reaction intermediates, respectively^55,56^, while the stretching mode of C=O is also observed at ~1677 cm^−1^ ^57,58^. Their intensity steadily grows at voltages from 0 to −0.5 V and is modestly evolving afterwards, which reflects the successful C−N coupling and the production of urea at this voltage range during the co-reduction of NO_3_^−^ and CO_2_.

To elucidate the changes in catalytic centre on NC during the reduction reactions, we investigate the evolution of N−H species in individual NtrRR by means of operando ATR-SEIRAS. To minimize the influence from NH_3_/N_2_H_4_ products, the potential is scanned from 0 to −0.15 V; at more negative voltages the formation of NH_3_/N_2_H_4_ will be initiated. We stress that while positive peaks in an ATR-SEIRAS spectrum represent an increase in concentration of the corresponding species at or near the surface, negative peaks can be ascribed to a decrease in concentration of the existing species. As shown in Fig. 5b, a negative peak appears at 3080 cm^−1^ that is attributed to N−H stretching vibration^59–62^, and its intensity is enhanced at more cathodic potentials. This suggests that the protons on existing C=N−H species at NC catalyst are diminishing at an initial stage of NtrRR, consistent with previous studies demonstrating the easy cleavage of N−H bonds on NC^36,37^. Therefore, we can infer that the NtrRR process directly consumes the protons on C=N−H species. Such a change in the catalytic centre is expected to produce an alteration in activity, thus creating the opportunity to switch the reaction path to CO_2_RR and enable the sequential mechanism for NO_3_^−^ and CO_2_ co-reduction to urea. After the formation of urea, the high thermodynamic driving force as mentioned above (∆*E*_2H_) could trigger rehydrogenation of NC, and the C=N−H species can serve as dynamically evolving active centres in the co-reduction reaction. We note that this N−H depletion feature is barely discernible on Cu_1_/NC in operando ATR-SEIRAS (Supplementary Figs. 39 and 40).

In situ Raman spectroscopy was also conducted to monitor the surface species during the catalytic reaction. As shown in Fig. 5c, a peak at 1050 cm^−1^ corresponding to the symmetrical stretching mode of nitrate ion^63,64^ is observed. The peaks that appeared at 1170 and 1417 cm^−1^ could be assigned to the stretching vibration of −NH_2_ and C−N, respectively^65,66^, indicating their formation in the co-reduction of NO_3_^−^ and CO_2_. ^15^N-isotope and ^13^C-isotope-labelled experiments (Fig. 5d) were performed under an applied potential of −0.5 V versus RHE with ^15^NO_3_^−^ and ^13^CO_2_ as the electrolyte and feeding gas. The positions of D and G bands of NC remain unchanged regardless of the isotope used. The Raman peak of −NH_2_ band shows a red shift in ^15^NO_3_^−^ isotope substitution experiments, and the peaks of ^12^C−^15^N, ^13^C−^14^N, ^13^C−^15^N are evidently shifted to lower values as compared to ^12^C−^14^N. These results verify that both nitrogen and carbon sources of urea originate from the employed feedstocks rather than from the functional groups on NC catalyst.

### DFT calculations of sequential reduction mechanism

**Fig. 6:**
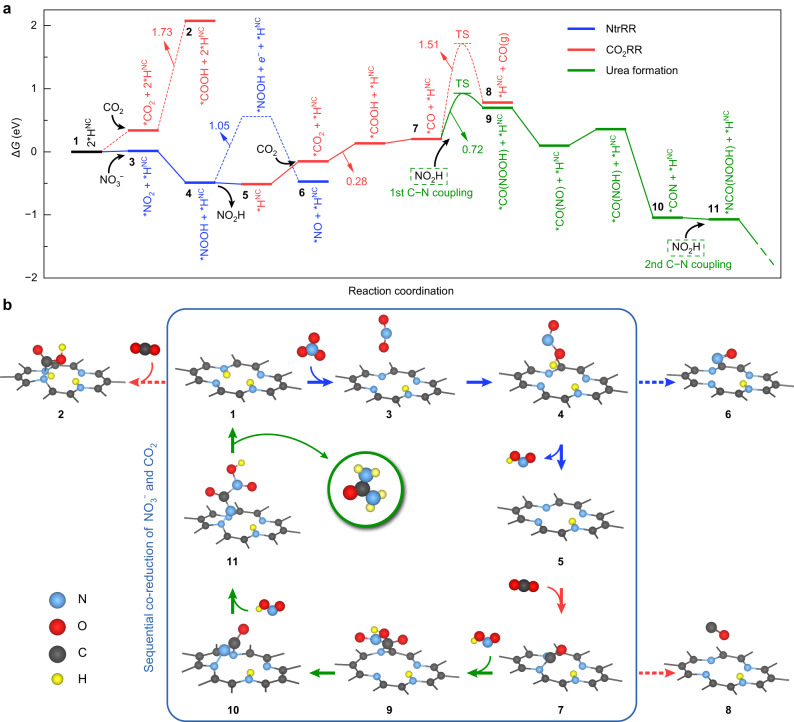
Proposed reaction path of the sequential co-reduction reaction on NC. **a** The DFT-calculated Gibbs free energy profile of the co-reduction reaction of NO_3_^−^ and CO_2_ at 0 V versus RHE. Elementary steps marked by dashed lines are less favourable than those marked by solid lines. The transition states (TS) of *CO desorption (**7** → **8**) and C−N coupling (**7** → **9**) were obtained by CI-NEB calculations. **b** Structural configurations of the critical reaction intermediates. Intermediates in the frame are related to the sequential co-reduction mechanism leading to urea formation.

Leveraging DFT Gibbs free-energy calculations, we can address the reaction pathway of urea electrosynthesis on the NC catalyst, which lends strong support to the sequential reduction scenario. Hydrogenated pyridinic N_4_ site with two bonded H atoms (configuration **1** in Fig. 6, denoted as 2*H^NC^) was taken as the representative catalytic centre. At the beginning, NtrRR will readily take place while CO_2_RR is disfavoured because of the significant barrier to form *COOH (**2**). Although NO_3_^−^ can be reduced into *NO_2_ in an electrochemical step (NO_3_^− ^+ 2H^+^ + e^−^ → *NO_2_ + H_2_O), this conversion can also occur via proton-coupled electron transfer from the catalyst (NO_3_^− ^+ *H^NC^ → *NO_2_ + OH^−^). In other words, NO_3_^−^ derives a proton-electron pair from the dehydrogenation of the N_4_ site, thus leaving one H^NC^ on the N_4_ site and forming a configuration of *NO_2_ + *H^NC^ (**3**), which is endothermic by only 0.08 eV. We find that the *NO_2_ intermediate cannot chemically bind to the catalyst, but is in fact trapped by van der Waals forces and located at a distance of around 3 Å from the catalytic centre (Supplementary Fig. 41). Similarly, *NOOH (**4**) and *NO (**6**) are physically rather than chemically adsorbed to the catalyst. The freestanding nature of these intermediates has led to a low overlap of electronic states between the adsorbed molecules and the catalyst. In particular, an energy gap of 1.05 eV exists between the lowest unoccupied molecular orbital (LUMO) of *NOOH and the Fermi level in configuration **4** (Supplementary Fig. 42). This result indicates that substantial energy is required to inject an electron from the catalyst to the adsorbed *NOOH molecule, thus imposing severe constraints on its electrochemical reduction. In comparison, the desorption process of *NOOH (**4**) is energetically favourable, exothermic by −0.05 eV, which offers ample opportunity for exposing the N_4_ site with one H^NC^ atom remaining (**5**). The easy desorption and migration of NO_2_H found by DFT calculations are in line with the experimental observation that NC catalyst is selective towards NO_2_^−^ formation during individual reduction of NO_3_^−^.

Subsequently, from configuration **5**, the reduced steric effect at the catalytic centre due to a missing H^NC^ is at play to guarantee facile CO_2_RR and C−N coupling processes. The steric effect gives rise to a strong interaction between *COOH and NC catalyst (Supplementary Fig. 43), which is the reason for the remarkable reduction in energy consumption at the *CO_2_ → *COOH step with one H^NC^ (0.28 eV) as compared to that with two H^NC^ atoms (1.73 eV). The high propensity for CO_2_RR at this stage supports our speculation that CO_2_ conversion can be promoted after NtrRR. The next bifurcation of the reaction pathway occurs at *CO, which will undergo either desorption or C−N coupling. Since both steps are kinetics-dominated processes, we here employed climbing-image nudged elastic band (CI-NEB)^67^ calculations (Supplementary Fig. 44) to determine their activation barriers. The results show that the barrier for the formation of CO(g) (**8**) is substantially higher than that of *CO(NOOH) (**9**), well matching with the inferior selectivity to CO product in the experiments. Then, the *CO(NOOH) intermediate undergoes three sequential electrochemical reduction steps and releases two water molecules to form *CON (**10**), at which the second C−N coupling takes place utilizing another NO_2_H nearby. The resultant intermediate *NCO(NOOH) (**11**) will undergo reduction into urea, and all the elementary steps in between are thermodynamically spontaneous (Supplementary Fig. 45) except for the *NCO(NO) → *NCO(NOH) step (0.34 eV). After the urea molecule leaves the catalyst surface, the N_4_ site will be hydrogenated immediately, leading to the regeneration of the 2*H^NC^ (**1**) configuration (Supplementary Fig. 17).

Collectively, the DFT results in Fig. 6 provide us with a comprehensive picture of the sequential reduction mechanism on NC. At the core of this mechanism lies the capability of structural alteration of the catalytic centre (between **1** and **5**), which is dynamic and reversible in the urea formation process. For comparison, DFT calculations were also performed for Cu_1_/NC. The Cu-N_4_ moiety corresponds to the catalytic centre, which may not undergo hydrogenation due to the saturation of all N atoms. The NtrRR process is initiated by the electrochemical reduction of NO_3_^−^, followed by further reduction into *NO with a flattened energy landscape (Supplementary Fig. 48). We note that unlike on NC catalyst, all the intermediates on Cu_1_/NC are chemically bonded to the catalytic centre (Supplementary Fig. 49), thus avoiding the exposure of the catalytic centre to other reactants. NtrRR continues to proceed until the formation of NH_3_ is completed, with a limiting potential of 0.31 V. On the other hand, the CO_2_RR process is sluggish due to the highly endothermic *CO_2_ → *COOH step (1.59 eV), similar to the case of N_4_ site with two H^NC^ atoms. As the Cu_1_/NC catalyst remains intact throughout the reactions, an equally huge amount of free energy input would be required to activate this step and afford the *CO intermediate, which is indispensable for urea formation. This suggests that NH_3_ is the most accessible product in the co-reduction of NO_3_^−^ and CO_2_ on Cu_1_/NC, showing good agreement with the experimental results. The proposed rationalization may also provide an explanation for the change in product selectivity on Cu_1_/NC: the overall >90% NH_3_ selectivity observed experimentally at −0.7 V is correlated with the competition for catalytic centres between NtrRR and CO_2_RR, while less negative potentials would lead to less fierce competition, and therefore more ample opportunities for concomitant reduction of both reactants.

## Discussion

In this work, selective urea electrosynthesis via a sequential reduction process utilizing NO_3_^−^ and CO_2_ as reactants is proposed and demonstrated on an N-doped carbon catalyst. This NC catalyst delivers a urea yield rate of 596.1 μg mg^−1^ h^−1^ with a high FE of 62% at −0.5 V versus RHE, outperforming most of the previously reported catalysts and showing great potential in large-scale application. In sharp contrast with NC, the Cu_1_/NC single-atom catalyst displays a much lower urea selectivity, which originates from the fierce and constant competition between the reduction of NO_3_^−^ and CO_2_ at a single catalytic centre. Based on rationally designed experiments, operando measurements and DFT calculations, we reveal the essential role of the C=N−H species on NC that could serve as dynamic active sites. The reaction preference is inherently controlled by the number of N−H bonds and is switchable between favouring NtrRR process or CO_2_RR process, whereby the reduction reactions are sequential and steered towards urea formation. The fundamental understanding of the sequential reaction model can form the basis for furthering the development of selective electrocatalysts to permit facile C−N coupling and efficient synthesis of urea.

## Methods

### Synthesis of NC and Cu_1_/NC

NC was prepared via a two-step pyrolysis method, using glucose (GC) and dicyandiamide (DCDA) as carbon source and nitrogen source, respectively. GC and DCDA were purchased from Sigma-Aldrich, and were directly used without further treatment. GC was mixed with DCDA at a mass ratio of 1: 40, and the mixture was sintered under flowing Ar at 550 °C for 6 h to form C_3_N_4_. Subsequently, the resultant product was carbonized at 900 °C for 3 h in an Ar atmosphere. The process of preparing Cu_1_/NC and other single-atom catalysts was the same as that of NC, except for adding the metal precursors such as Cu(NO_3_)_2_. Finally, aggregates of metal particles were removed by washing the catalysts thoroughly in 4.0 M H_2_SO_4_.

### Ex situ and in situ characterizations

The XRD patterns were collected on a Bruker D8 Advance diffractometer equipped with Cu Kα radiation, scanning from 10° to 80° with a scan rate of 2° min^−1^. The structure and morphology of the samples were investigated on a field emission scanning electron microscope (ZEISS SUPRA®55, Carl Zeiss) and a transmission electron microscope (JEM–3200FS, JEOL) equipped with an EDS detector. XPS experiments were conducted on an ESCALAB 250X instrument (Thermo Fisher).

The electrochemical operando ATR-SEIRAS was measured by INVENIO R FTIR spectrometer (Bruker) equipped with a mercury-cadmium-telluride (MCT) detector. An Ag/AgCl electrode and a Pt foil were employed as the reference electrode and the counter electrode, respectively. During spectrum collection, the optical path was continuously purged with nitrogen gas to minimize the disturbance caused by water and CO_2_ in the air. A CO_2_-saturated electrolyte containing 0.1 M KHCO_3_ and 0.1 M KNO_3_ was employed in the co-reduction reaction process of NO_3_^−^ and CO_2_. The background spectrum of the catalyst electrode was obtained at an open-circuit potential before each measurement. Then, the absorbance spectra (−log(R/R_0_)) at different potentials were collected at a spectral resolution of 4 cm^−1^. In the ATR-SEIRAS spectra, a negative peak indicates that a certain substance or functional group is consumed, while a positive peak indicates that a certain substance or functional group is produced or has increased.

The in situ Raman characterization was performed in a three-electrode cell equipped with a confocal microscope Raman system (Renishaw inVia). The wavelength was 532 nm with a 50× microscope objective. During the testing process, the distance between the sapphire window and the electrode was less than 0.1 nm, which ensures that the attenuation effect of the solution layer on Raman signal can be as small as possible.

### Electrochemical measurement

The electrochemical measurement was conducted on a three-electrode configuration using a CHI 660E electrochemical station in the H-type cell. Nafion 115 (Dupont) was pretreated and used to assemble the H-type cell. The Nafion membrane was first heated in 5% H_2_O_2_ at 80 °C for 1 h and rinsed by deionized water. Then, it was further heated in 5% H_2_SO_4_ at 80 °C for 1 h and rinsed by deionized water again. An electrolyte composed of 0.1 M KHCO_3_ + 0.1 M KNO_3_ and saturated with CO_2_ (pH = 6.8) was employed, with an Ag/AgCl electrode (in saturated KCl) and a platinum foil (1 cm × 1 cm) used as the reference electrode and the counter electrode, respectively. 1 mg of catalyst material was dispersed in 900 μL deionized water, 50 μL isopropanol and 50 μL Nafion (5 wt% aqueous solution), and then sonicated for 3 h under ice-water bath. Afterwards, 150 µL of the catalyst ink was loaded onto a carbon paper (Toray, TGP-060) and dried in the ambient environment to form the working electrode (geometric area: 1 × 1 cm^2^; mass loading: 0.15 mg cm^−2^). Before electrochemical tests, CO_2_ was purged into the electrolyte for 30 min with a flow rate of 100 sccm to remove the residual air. Potentiostatic electrolysis for 30 min with a CO_2_ flow rate of 20 sccm was done at each potential, and the gas products were analysed with a 10-min interval by gas chromatography (SHIMADZU, GC-2014C) equipped with a flame ionization detector (FID) and a thermal conductivity detector (TCD). The gas chromatography was calibrated using standard samples under standard conditions (1 atm, 298 K). The linear sweep voltammetry test was performed at a scan rate of 10 mV s^−1^. The N-selectivity of reaction products was calculated as follows:

1 $${S}_{{{{{{\rm{N}}}}}}-{{{{{\rm{selectivity}}}}}}}=\frac{M(N)}{{M}_{{{{{{\rm{total}}}}}}}(N)}*100\%$$

where *M*(*N*) and *M*_total_(*N*) are the moles of nitrogen for a specific N-containing product and for all products in NtrRR, respectively.

### Computational details

All the theoretical calculations were performed via spin-polarized DFT using Vienna ab initio simulation pack (VASP 5.3.5) with projector augmented wave method^68^. The Perdew−Burke−Ernzerhof (PBE) exchange-correlation functional was employed along with a plane-wave cutoff energy of 450 eV. The third-generation (D3) semi-empirical van der Waals corrections proposed by Grimme^69^ were adopted in structural optimization to deal with the dispersion interactions. Supercells consisting of 5 × 5 unit cells were constructed for the carbon layers of both NC and Cu_1_/NC catalysts, with a vacuum space of >20 Å to separate the slabs in the *z* direction. The Brillouin zone was sampled by Γ-centred Monkhorst–Pack *k*-point mesh of 2 × 2 × 1 for free energy calculations. To take into account the contribution of hydrogen bonds, we introduced four water molecules in our simulations for the adsorbate configurations. Each image in the climbing-image nudged elastic band (CI-NEB) calculations was relaxed until a force tolerance of 0.02 eV/Å was reached.

Computational hydrogen electrode (CHE) model^70^ was employed for Gibbs free energy calculation in every elementary step. In this scheme, the free energy of H^+^/*e*^–^ pair is equivalent to the chemical potential of gaseous H_2_ at standard conditions (pH = 0, *P* = 1 bar, *T* = 298 K). The free energy change Δ*G* of each elementary step can be calculated by the following equation:

2 $$\Delta G=\Delta E+\Delta {E}_{{{{{{\rm{ZPE}}}}}}}-T\Delta S+{eU}+{k}_{{{{{{\rm{B}}}}}}}T{{{{\mathrm{ln}}}}}(10)\times {{{{{\rm{pH}}}}}}$$

where *E* is the total energy directly calculated by DFT; *E*_ZPE_ is the zero-point energy, which was obtained from the calculated vibrational frequencies of the adsorbates using normal mode analysis; *S* is the entropy and *T* was set to room temperature (298 K); *U* is the potential measured against the standard hydrogen electrode; *k*_B_ is Boltzmann constant. The entropy for each reaction intermediate was calculated by the following equation:

3 $$S=\mathop{\sum }\limits_{i}\frac{\frac{h{v}_{i}}{T}}{{e}^{\frac{h{v}_{i}}{{k}_{{{{{{\rm{B}}}}}}}T}}-1}-{k}_{{{{{{\rm{B}}}}}}}\mathop{\sum }\limits_{i}{{{{\mathrm{ln}}}}}\left(1-{e}^{\frac{-h{v}_{i}}{{k}_{{{{{{\rm{B}}}}}}}T}}\right)$$

where *h* and *v*_*i*_ are Planck constant and the vibrational frequencies of the adsorbates, respectively. In this work, the free energy profiles were calculated at 0 V versus RHE and pH = 6.8.

### Supplementary information

Supplementary Information

Peer Review File

### Source data

Source Data

## Data Availability

The data generated or analysed during this study are included in this published article and its supplementary information files, and are available from the corresponding authors on reasonable request. Source Data are provided with this paper.
