# Supplementary material for: Sequential co-reduction of nitrate and carbon dioxide enables selective urea electrosynthesis
Source: Nat Commun. 2024 Jan 2;15:176. doi: 10.1038/s41467-023-44131-z (PMC10761727; doi:10.1038/s41467-023-44131-z)
Supplement: Supplementary file 1 — Supplementary Information [file 41467_2023_44131_MOESM1_ESM.pdf]

Supplementary Information

Sequential co-reduction of nitrate and carbon dioxide enables selective urea electrosynthesis

Yang Li[1,2,6], Shisheng Zheng[1,6], Hao Liu[1], Qi Xiong[1], Haocong Yi[1], Haibin Yang[1], Zongwei Mei[1], Qinghe Zhao[1], Zu-Wei Yin[1], Ming Huang[*,3], Yuan Lin[4], Weihong Lai[5], Shi-Xue Dou[5], Feng Pan[1], Shunning Li[*,1]

[1]School of Advanced Materials, Peking University, Shenzhen Graduate School, Shenzhen, Guangdong 518055, China

[2]Hydrogen Energy Institute, Zhejiang University, Hangzhou, Zhejiang 310027, China

[3]Institute of Fundamental and Frontier Sciences, University of Electronic Science and Technology of China, Chengdu 611731, China

[4]Institute of Chemistry, Chinese Academy of Sciences, Beijing 100190, China

[5]Institute for Superconducting and Electronic Materials, University of Wollongong, Wollongong, NSW 2522, Australia

[6]These authors contributed equally to this work.

*Correspondence and requests for materials should be addressed to Ming Huang or Shunning Li.

***Email**: huangming@uestc.edu.cn (M.H.); lisn@pku.edu.cn (S.L.)

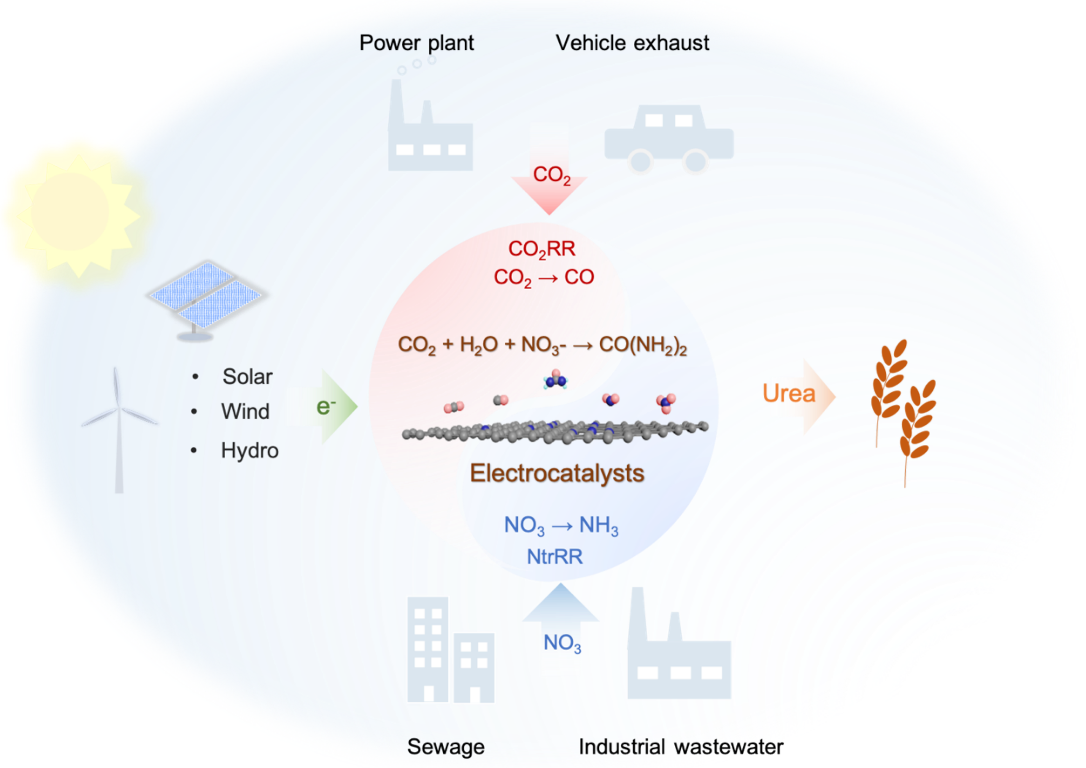

Supplementary Figure 1. Schematic illustration of urea electrosynthesis driven by renewable electrical energy.

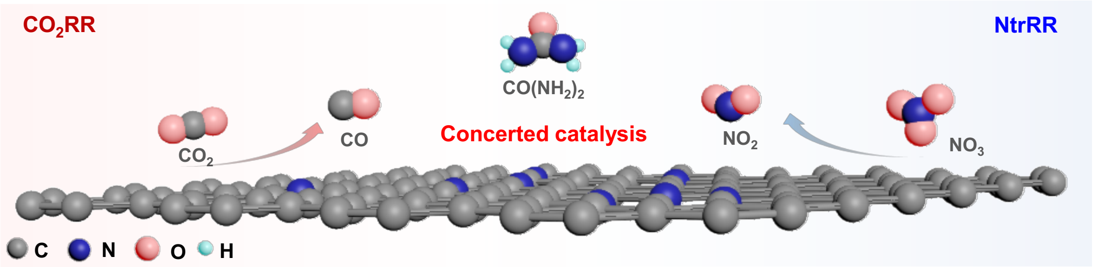

Supplementary Figure 2. Schematic illustration of coupling NO_3^- and CO_2 in water to synthesize urea.

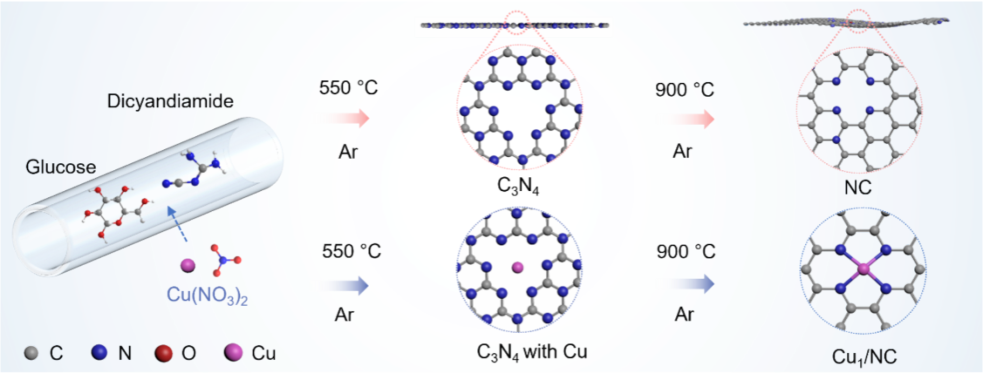

Supplementary Figure 3. Schematic illustration showing the preparation of N-doped carbon (NC) and Cu single atom dispersed N-doped carbon (Cu_1/NC).

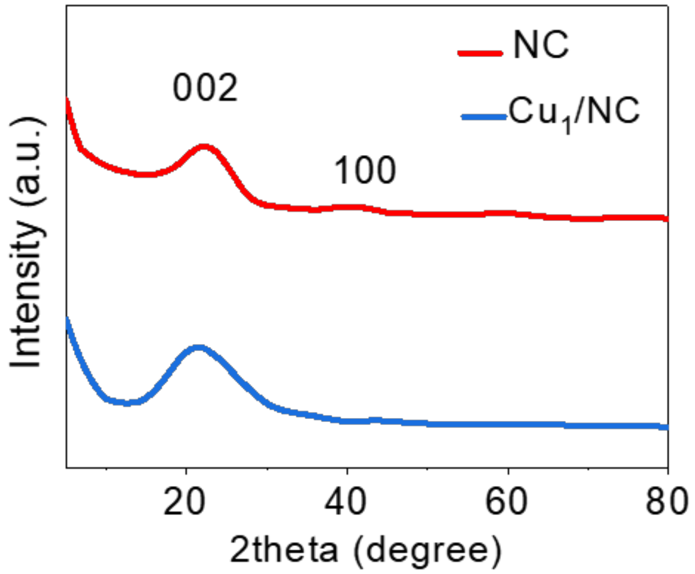

Supplementary Figure 4. XRD patterns of NC and Cu_1/NC.

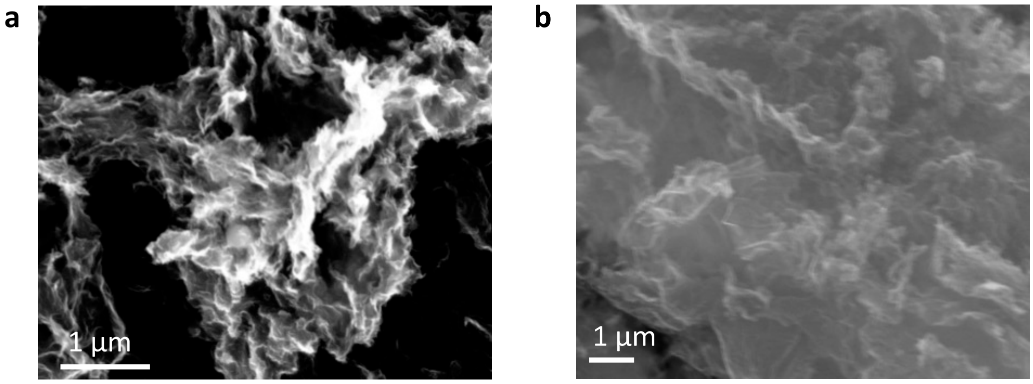

Supplementary Figure 5. SEM images of (a) NC and (b) Cu_1/NC.

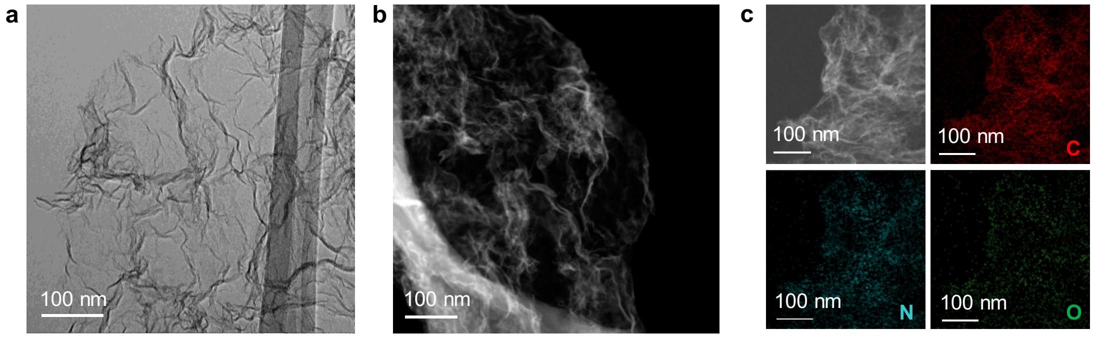

Supplementary Figure 6. Microstructural characterization of NC. (a) TEM image. (b) HAADF-TEM image. (c) EDS mapping.

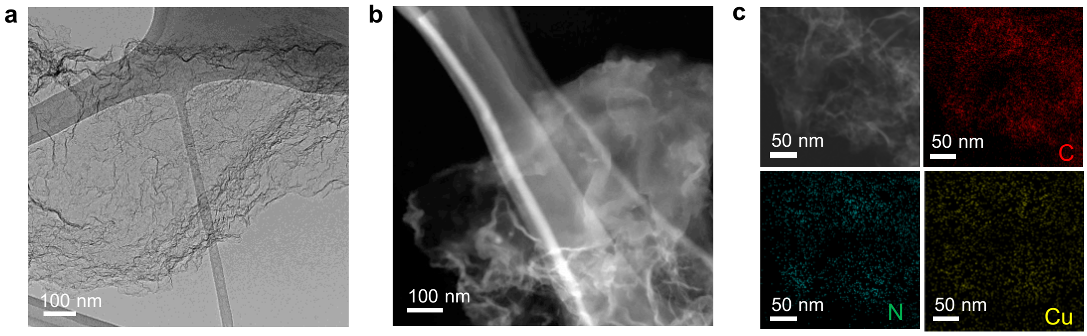

Supplementary Figure 7. Microstructural characterization of Cu_1/NC. (a) TEM image. (b) HAADF-TEM image. (c) EDS mapping.

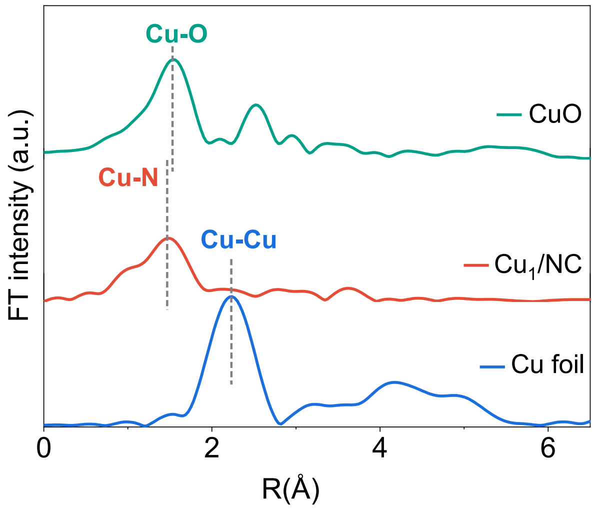

Supplementary Figure 8. EXAFS spectroscopy of Cu_1/NC.

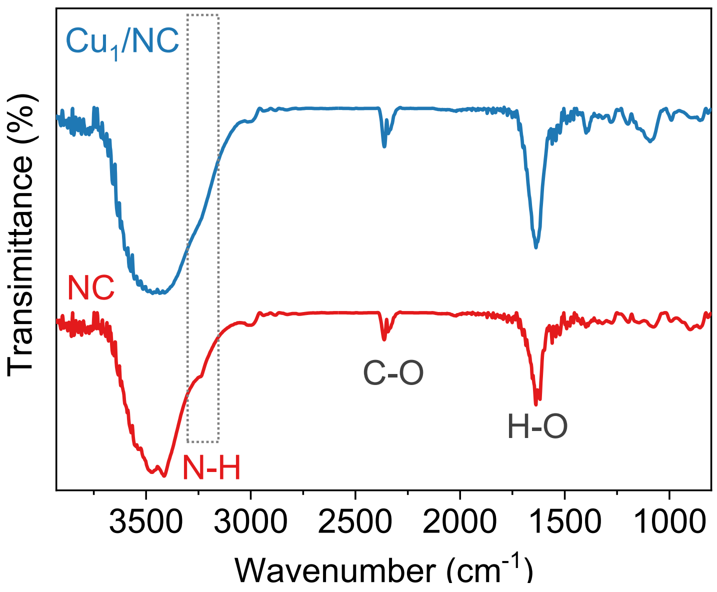

Supplementary Figure 9. FTIR spectra of NC and Cu_1/NC. The band at 3243 cm^{-1} corresponds to N−H stretching mode.

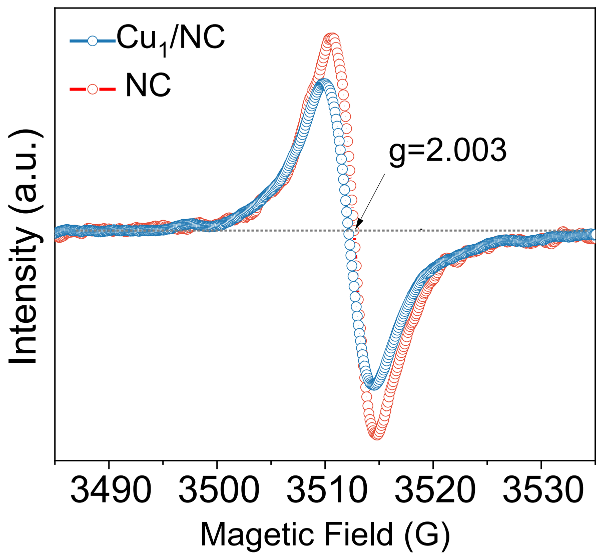

Supplementary Figure 10. EPR spectra of NC and Cu_1/NC. As the nitrogen atom can redistribute the extra electron to adjacent carbon via the delocalized π-conjugated network of carbon layer, an EPR signal at g = 2.003 observed in nitrogen-doped carbon can be assigned to the unpaired electron on the carbon atom. This result is well in line with Raman spectrum and STEM observation.[1]

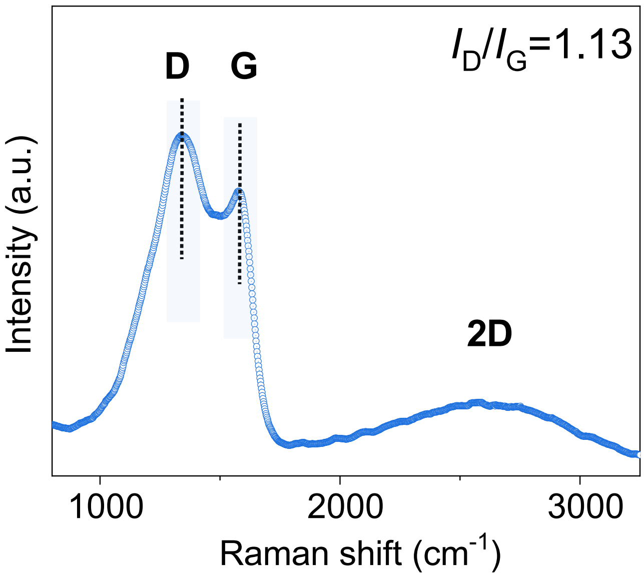

Supplementary Figure 11. Raman spectrum of NC. Two first-order Raman peaks centered at 1320 cm^{-1} and 1586 cm^{-1} can be assigned to D and G bands, respectively. The G band originates from the ordered sp^2 bonded carbon, representing the formation of graphitic carbon. The D band arises from the disordered sp^3 carbon in the defects. The ratio of integrated intensity of D and G bands (I_D/I_G) is ~1.13 for NC, indicating abundant structural defects and disordered carbon.[2]

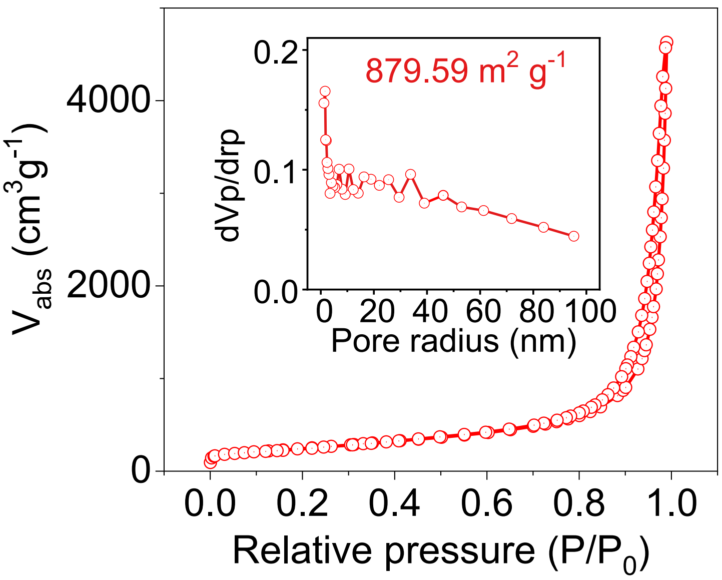

Supplementary Figure 12. N_2 adsorption and desorption isotherm for NC. The BET surface area of NC is ~879.59 m^2 g^{-1}. Inset: pore size distribution for NC.

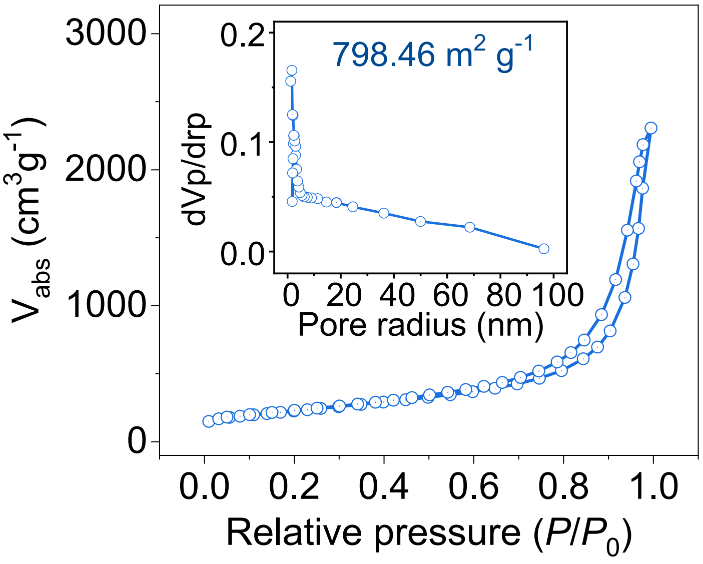

Supplementary Figure 13. N_2 adsorption and desorption isotherm for Cu_1/NC. The BET surface area of Cu_1/NC is ~798.46 m^2 g^{-1}. Inset: pore size distribution for Cu_1/NC.

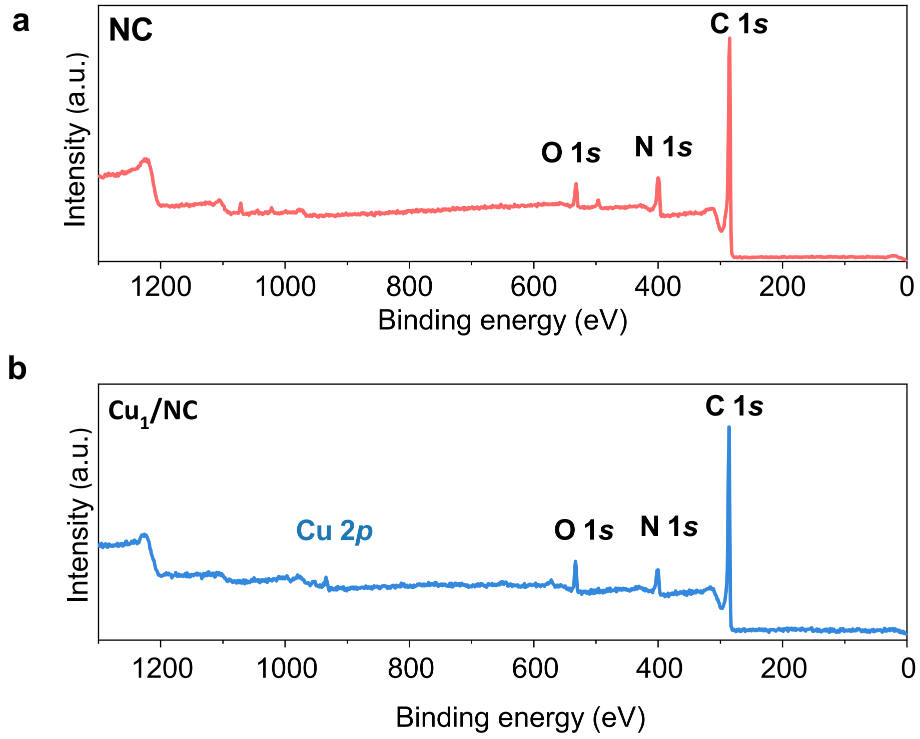

Supplementary Figure 14. XPS spectra of (a) NC and (b) Cu_1/NC.

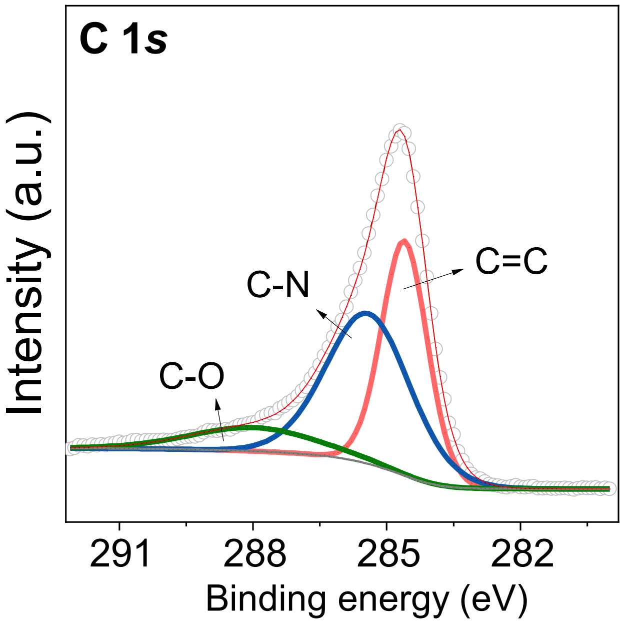

Supplementary Figure 15. C 1*s* XPS spectrum of NC. The spectrum can be deconvoluted into components of C=C, C−N and C−O.

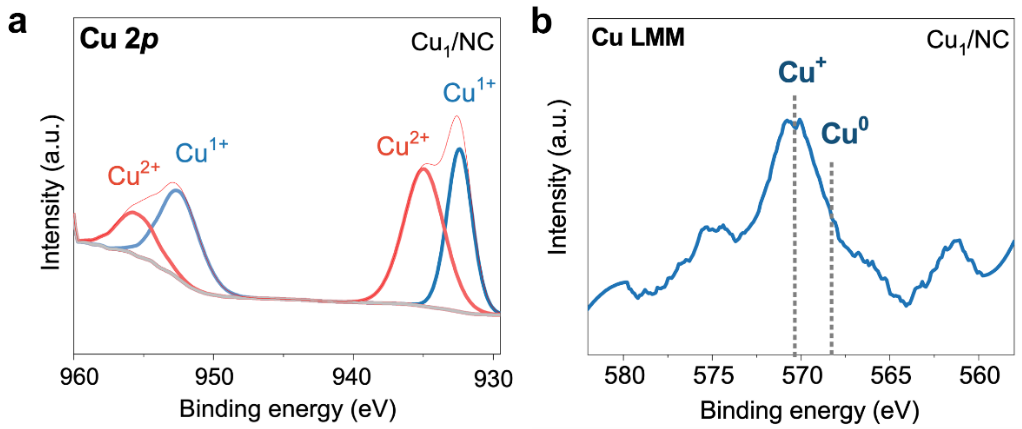

Supplementary Figure 16. (a) High-resolution Cu 2*p* XPS spectrum of Cu_1/NC. (b) Cu LMM spectrum of Cu_1/NC.

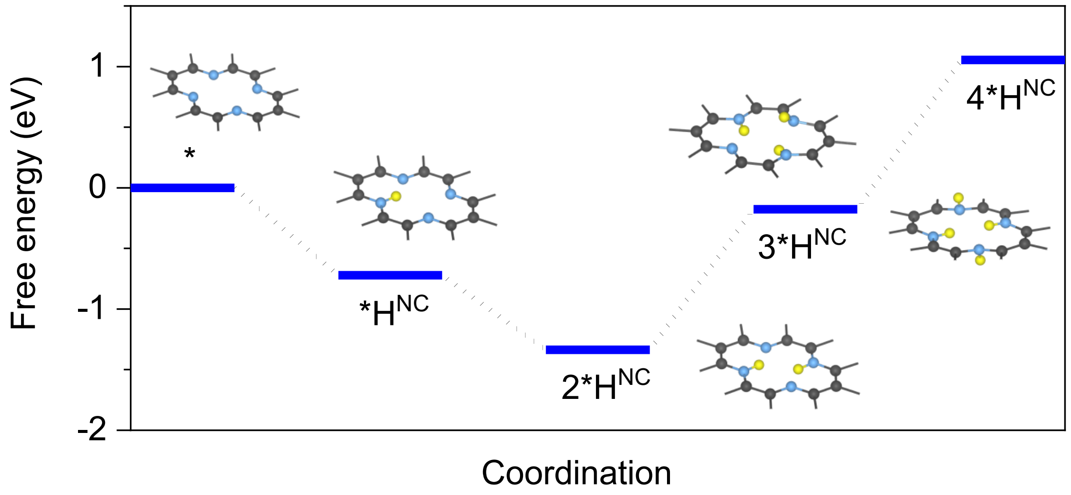

Supplementary Figure 17. Free energy diagram of hydrogen binding on NC at 0 V versus RHE, and the corresponding atomic structures. Color code: N, blue; C, black; H, yellow.

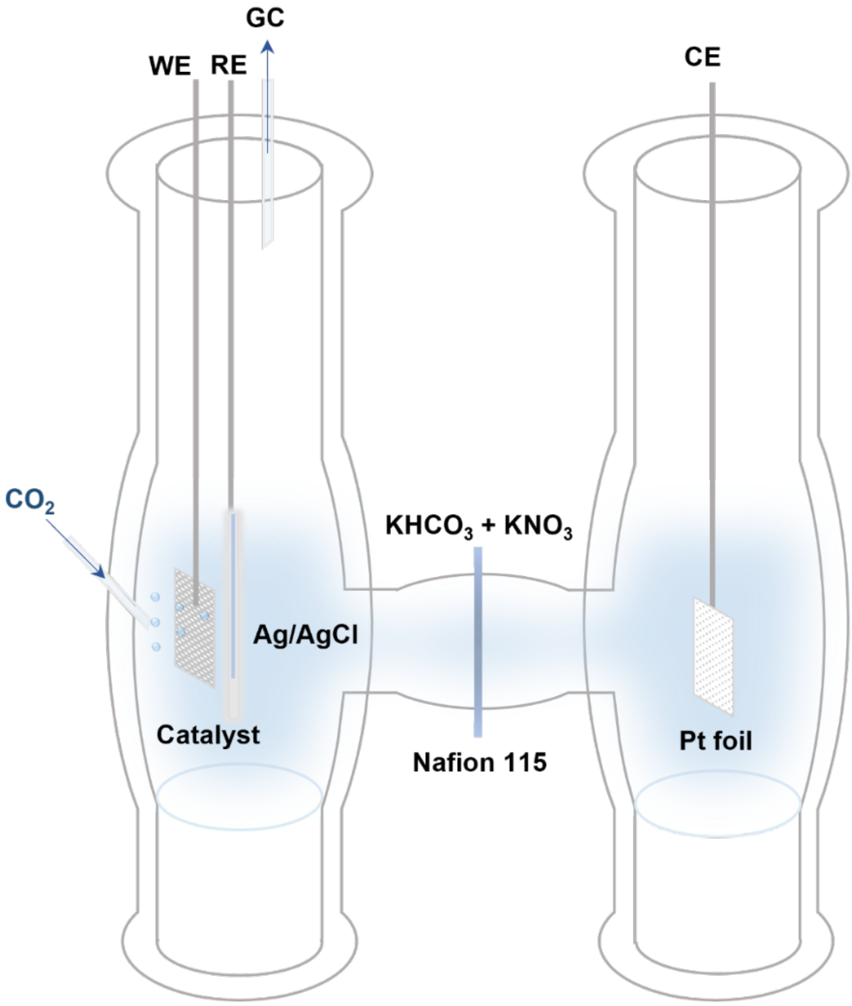

Supplementary Figure 18. Schematic illustration of the electrochemical configuration for urea synthesis.

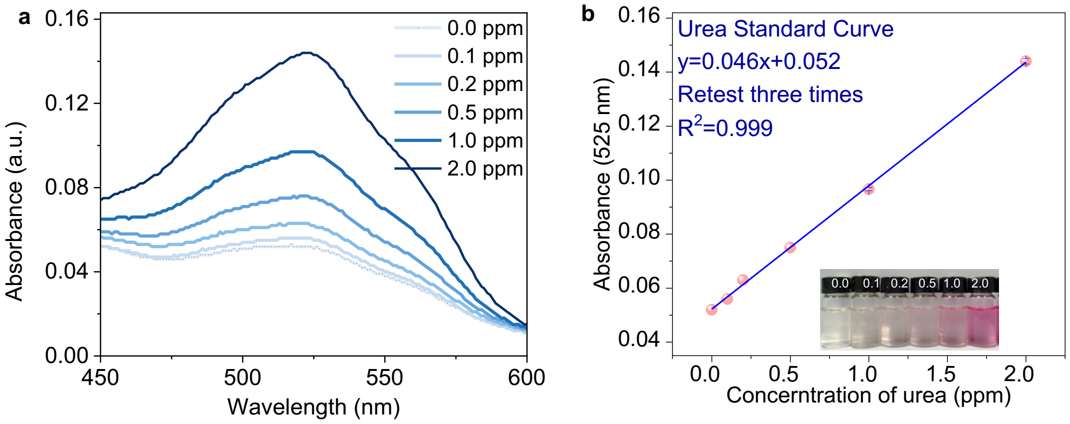

Supplementary Figure 19. Quantification of urea by the diacetyl monoxime method[3] in 0.1 M $KHCO_3$ and 0.1 M KNO_3 electrolyte. (a) UV-vis absorption spectra. (b) Standard curve of urea concentration.

The color reagents were prepared as follows:

***A*. Acid-ferric solution:** 50 mL of concentrated H_3PO_4,150 mL of concentrated H_2SO_4 and 300 mL of distilled water were mixed, and then 50 mg of $FeCl_3$ was dissolved in the above solution.

***B*. Diacetyl monoxime thiosemicarbazide (DAMO-TSC) solution:** 2.5 g of DAMO and 50 mg of TSC were dissolved in distilled water and diluted to 500 mL.

A series of standard urea solutions with concentrations of 0 ppm, 0.2 ppm, 0.5 ppm, 1.0 ppm, 2.0 ppm and 5.0 ppm in 0.1 M $KHCO_3$ and 0.1 M KNO_3 electrolyte were prepared. The following procedure was conducted: 1 mL of standard urea solution, 2 mL of acid-ferric solution and 1mL of DAMO-TSC solution were mixed and heated under 100 °C for 15 min. The absorbance measurement was performed at λ = 525 nm with an ultraviolet-visible spectrophotometer. The calibration curve (y = 0.260, x = 0.051, coefficient of determination, R^2= 0.999) showed a good linear relationship of absorbance values with the urea concentration in three independent calibrations. The inset shows the pink solutions with different urea concentrations.

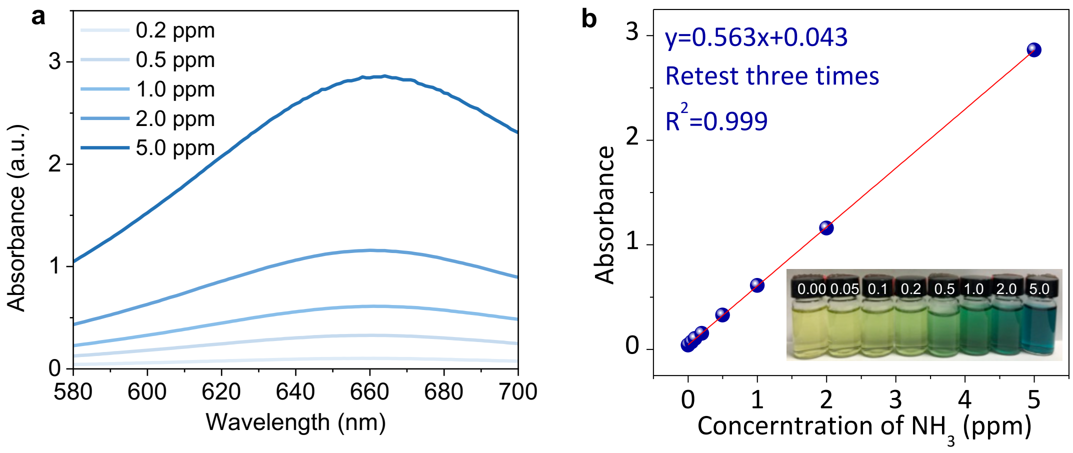

Supplementary Figure 20. Quantification of NH_3 by the indophenol blue method[4] in 0.1 M $KHCO_3$ and 0.1 M KNO_3 electrolyte. (a) UV-vis absorption spectra. (b) Standard curve of NH_3 concentration.

The color regents were prepared as follows:

***A*. Oxidation solution**: 0.75 M NaOH and sodium hypochlorite (available chlorine, 4.00-4.99%);

***B.* Coloring solution:** 0.4 M sodium salicylate and 0.32 M NaOH;

***C.* Catalyst solution:** 0.1 g $Na_2[Fe(NO)(CN)_5]\cdot 2H_2O$ diluted in 10 mL deionized water. A series of standard NH_4Cl solutions with concentrations of 0.0, 0.2, 0.5, 1.0, 2.0, 4.0, and 10.0 μg mL^{-1} in 0.1 M $KHCO_3$ and 0.1 M KNO_3 electrolyte were prepared. The following procedure was conducted: 4 mL standard solutions were separately mixed with 50 μL of oxidation solution, 500 μL of coloring solution, and 50 μL of catalyst solution. The absorbance measurement was performed at λ = 655 nm with an ultraviolet-visible spectrophotometer. The calibration curve (y = 0.260, x = 0.051, coefficient of determination, R^2 = 0.999) showed a good linear relationship of absorbance values with the NH_3 concentration in three independent calibrations. The calibration curve was used to calculate the NH_3 concentration. The inset shows the green solutions with different NH_3 concentrations.

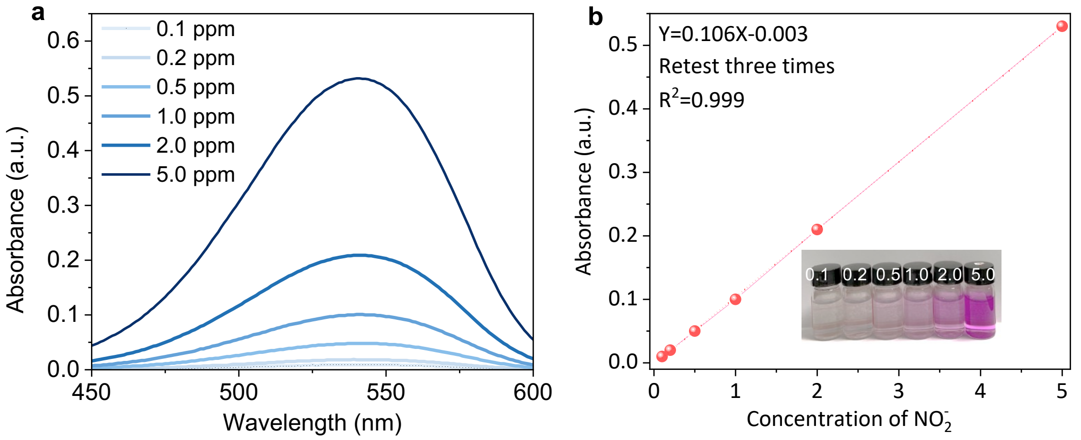

Supplementary Figure 21. Quantification of NO_2^- by Griess test[5] in 0.1 M $KHCO_3$ and 0.1 M KNO_3 electrolyte. (a) UV-vis absorption spectra. (b) Standard curve of NO_2^- concentration.

The Griess reagent was prepared as follows:

1.0 g of sulfonamide, 0.1 g of N-(1-naphthyl) ethyldiamine dihydrochloride, and 2.94 L of H_3PO_4 were mixed within 50 mL deionized water. Subsequently, a series of NO_2^- standard solution (0 ppm, 0.2 ppm, 0.5 ppm, 1.0 ppm, 2.0 ppm, 5.0 ppm) were prepared. For the colorimetric assay, 1.0 mL of NO_2^- solution, 1 mL of Griess reagent and 2 mL of H_2O were mixed and reacted for 10 min. The absorbance was measured by ultraviolet-visible spectroscopy at 540 nm.

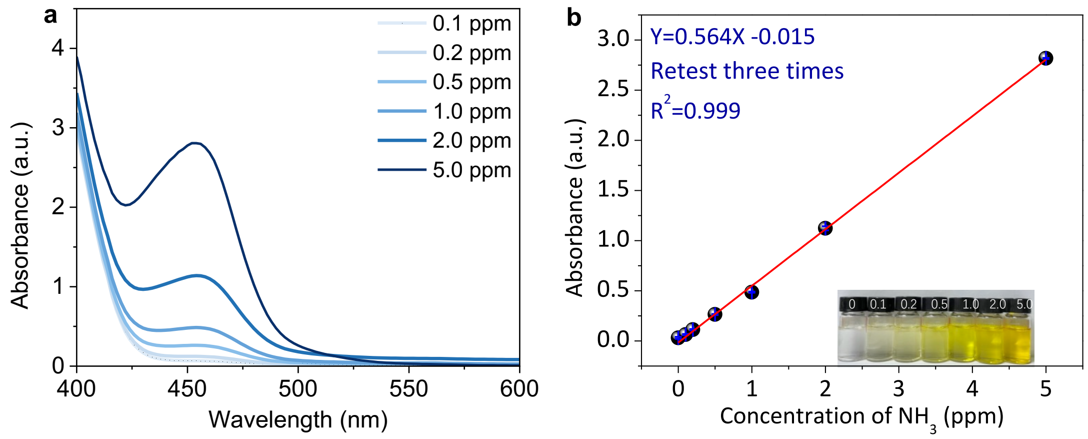

Supplementary Figure 22. Quantification of N_2H_4 by Watt and Chrisp's method[6] in 0.1 M $KHCO_3$ and 0.1 M KNO_3 electrolyte. (a) UV-vis absorption spectra. (b) Standard curve of N_2H_4 concentration.

The color reagent was prepared by dissolving para-(dimethylamine) benzaldehyde (5.99 g) with a mixture of 30 mL HCl (12 mol L^{-1}) and ethanol (300 mL). 2 mL of standard hydrazine solution in different concentrations was mixed with 2 mL of color regent. After 20 min, the absorbance of the colored solutions was measured at 455 nm by UV-vis. Then, 2 mL of the electrolyte after electrolysis was mixed with 2 mL of color regent.

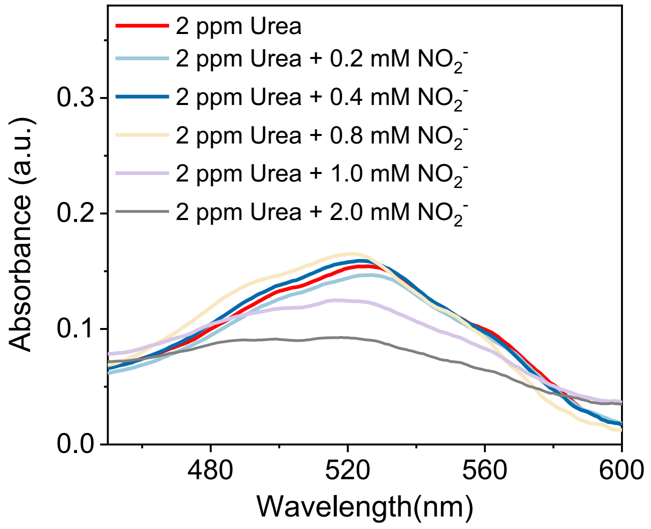

Supplementary Figure 23. UV-vis absorption spectra of standard solutions containing 2 ppm urea with various NO_2^- concentrations in DAMO-TSC measurements. The results indicate that the influence of NO_2^- on urea determination is trivial when the NO_2^- concentration is relatively low.

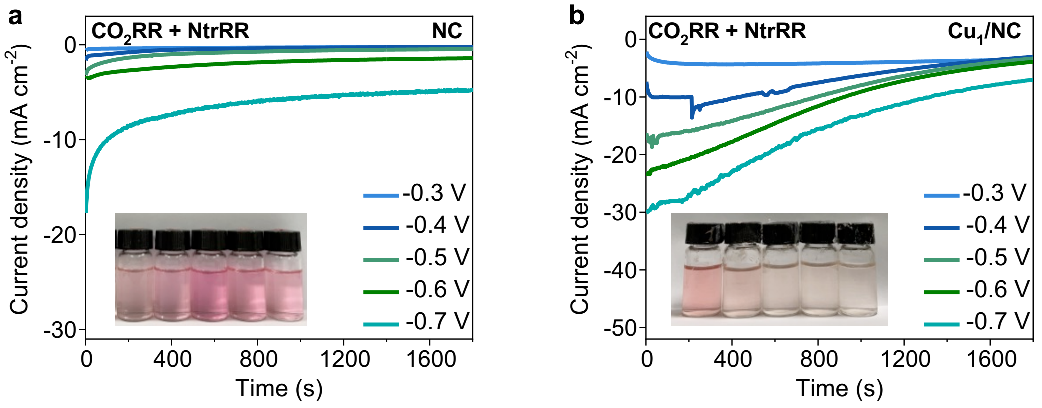

Supplementary Figure 24. Performance of urea synthesis on NC and Cu_1/NC. Chronoamperometric curves of (a) NC and (b) Cu_1/NC at different potentials. The insets show the color of the solutions after co-reduction.

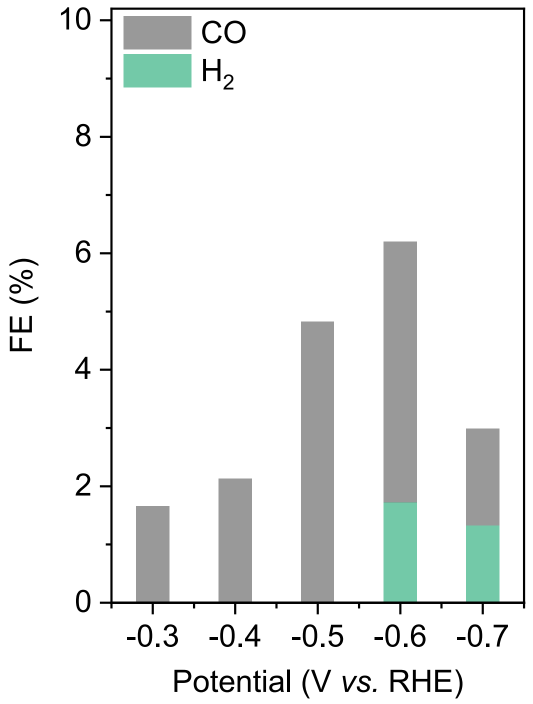

Supplementary Figure 25. Faradaic efficiencies of CO and H_2 in the co-reduction reaction on NC.

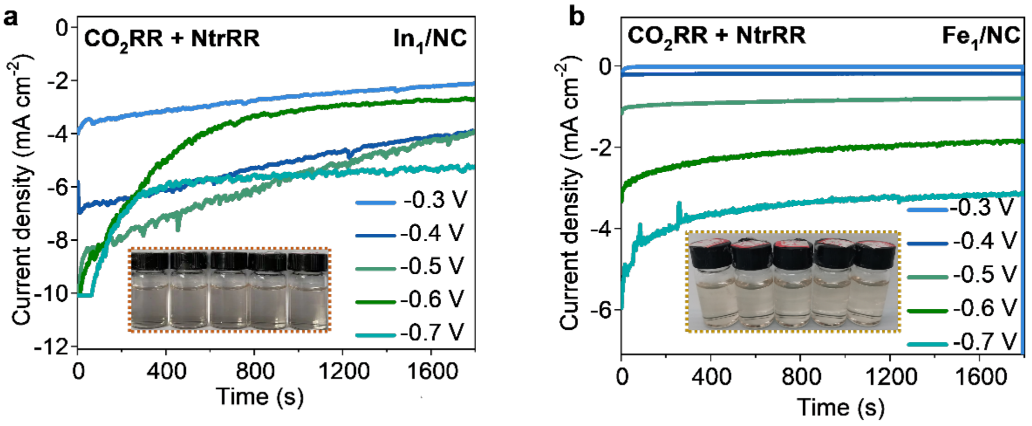

Supplementary Figure 26. Chronoamperometric curves of (a) In_1/NC and (b) Fe_1/NC under different applied potentials in the co-reduction reaction.

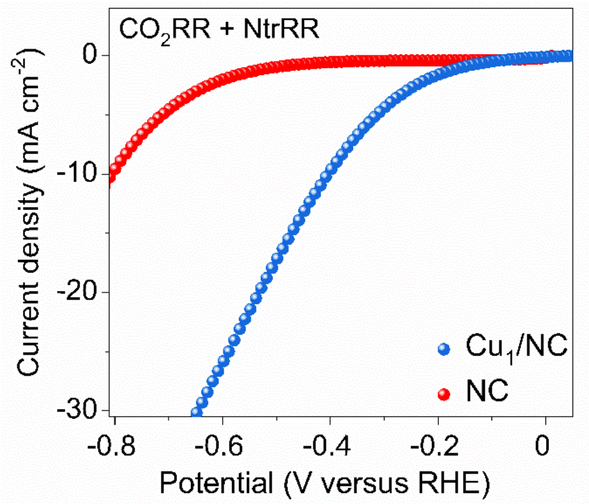

Supplementary Figure 27. Linear sweep voltammetry (LSV) curves of co-reduction reaction on NC and Cu_1/NC.

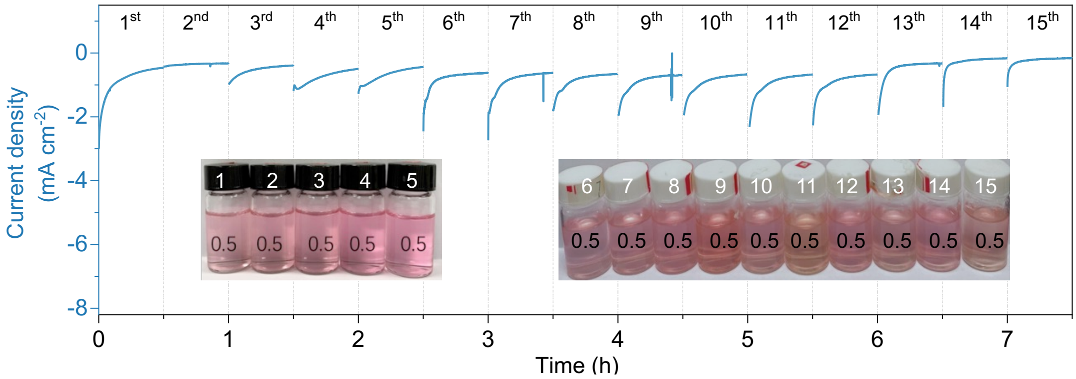

Supplementary Figure 28. Stability test of NC at −0.5 V versus RHE for 15 cycles. Performance was well maintained for the first 12 cycles. In the 13th cycle, some of the active materials were blown off by CO_2 gas, leading to sudden reduction in current density.

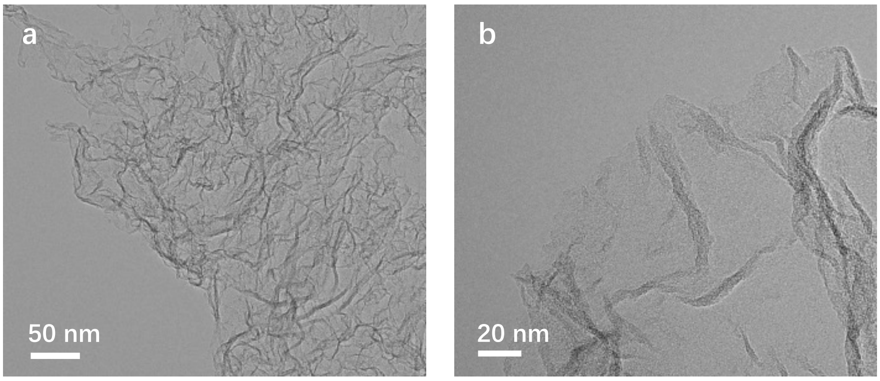

Supplementary Figure 29. TEM images of NC after the stability test with scale bars of (a) 50 nm and (b) 20 nm.

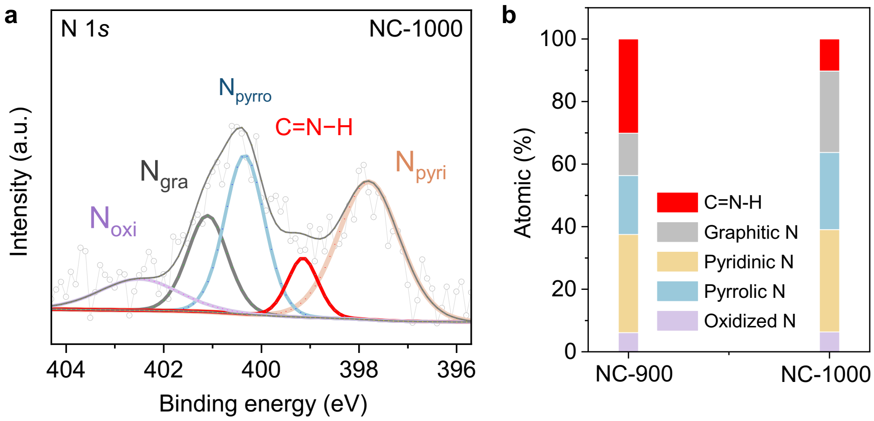

Supplementary Figure 30. (a) N 1*s* XPS spectrum of NC catalyst prepared at 1000 °C (NC-1000). (b) The ratios of C=N−H, graphitic N, pyridinic N, pyrrolic N and oxidized N in NC-900 (NC catalyst prepared at 900 °C) and NC-1000.

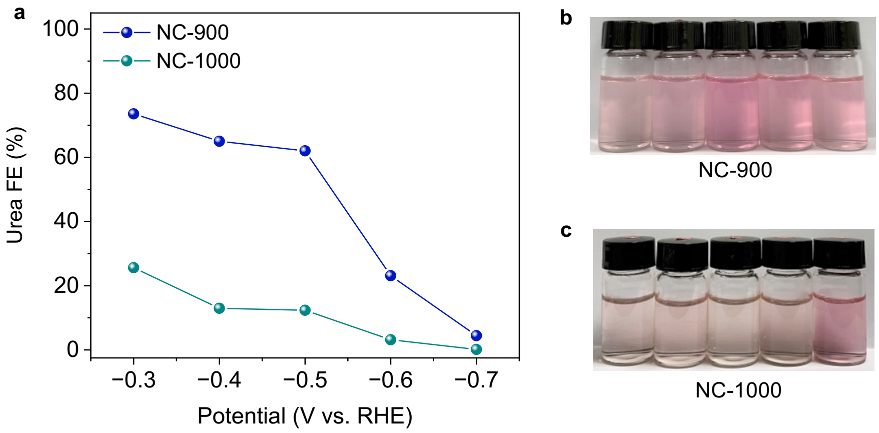

Supplementary Figure 31. (a) The FE of urea for NC-900 and NC-1000. (b and c) The color of the solutions after the co-reduction reaction on (b) NC-900 and (c) NC-1000.

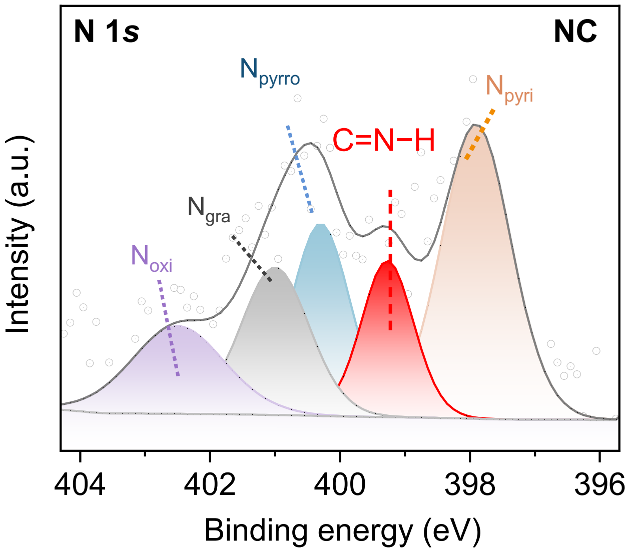

Supplementary Figure 32. N 1*s* XPS spectrum of NC after the stability test. Carbon paper at the working electrode will affect the accuracy of measurement to some extent.

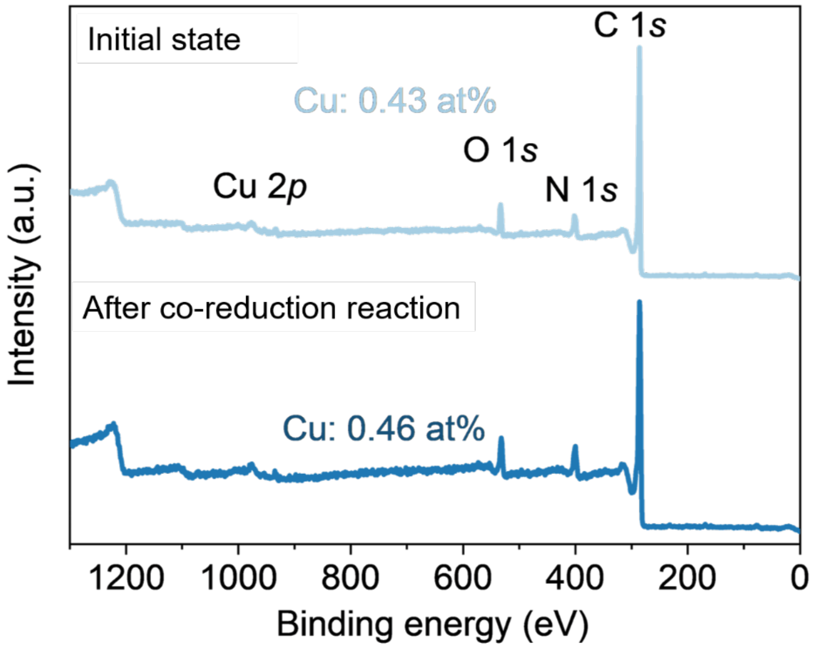

Supplementary Figure 33. XPS spectra of Cu_1/NC before and after the co-reduction reaction at −0.5 V versus RHE. The results indicate that the leaching of Cu atoms is negligible during the reaction. These XPS data were collected on carbon paper.

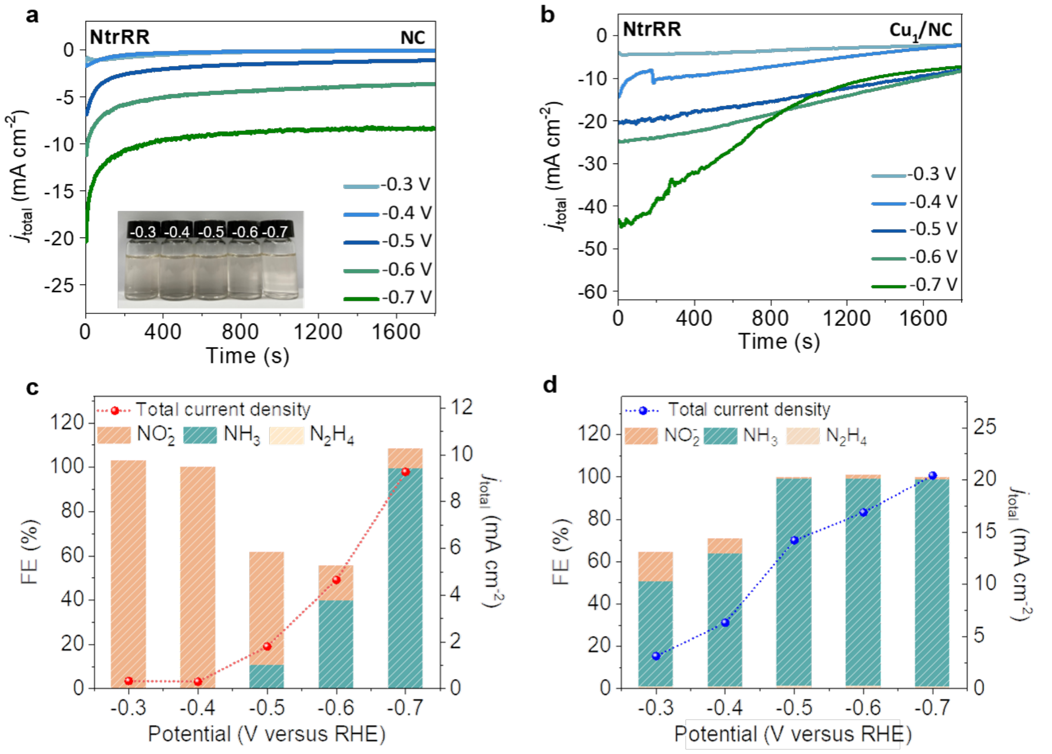

Supplementary Figure 34. Individual NtrRR experiments on NC and Cu_1/NC. (a and b) Chronoamperometric curves of (a) NC and (b) Cu_1/NC at different potentials. The inset in (a) shows that the color of the solution determined by the diacetyl monoxime method after individual NtrRR is unchanged, suggesting no urea formation during the NtrRR process over NC. (c and d) Faradaic efficiencies and total current densities on (c) NC and (d) Cu_1/NC at different potentials.

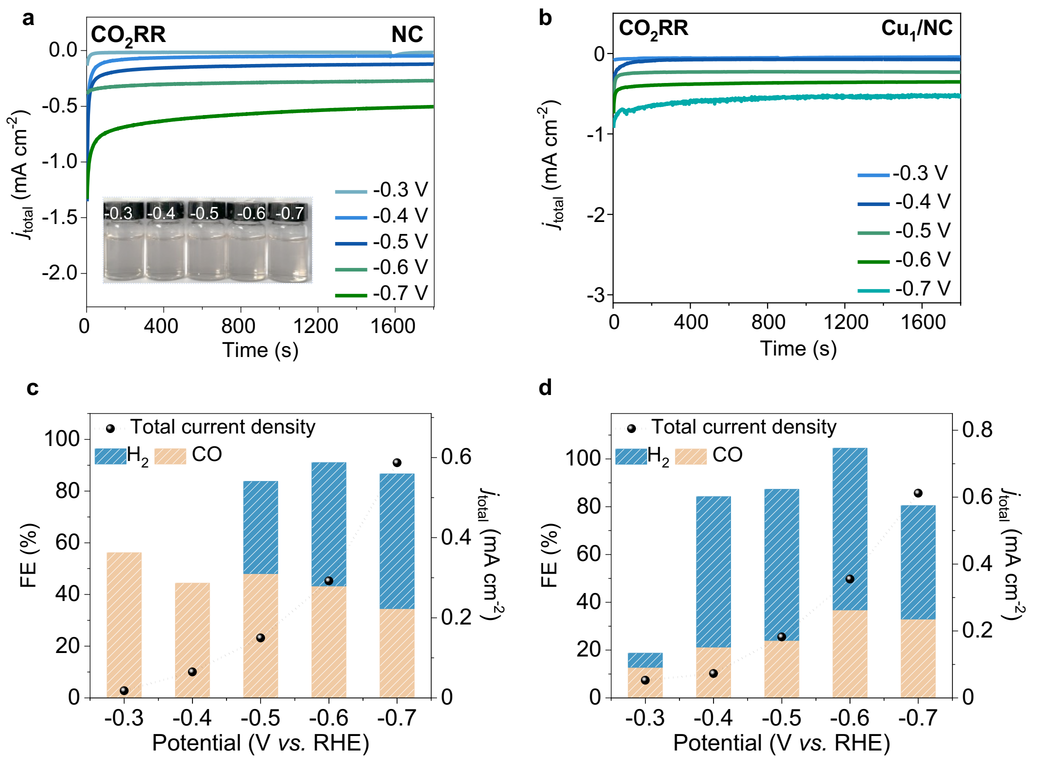

Supplementary Figure 35. Individual CO_2RR experiments on NC and Cu_1/NC. (a and b) Chronoamperometric curves of (a) NC and (b) Cu_1/NC at different potentials. The inset in (a) shows that the color of the solution determined by the diacetyl monoxime method after individual CO_2RR is unchanged, suggesting no urea formation during the CO_2RR process over NC. (c and d) Faradaic efficiencies and total current densities on (c) NC and (d) Cu_1/NC at different potentials.

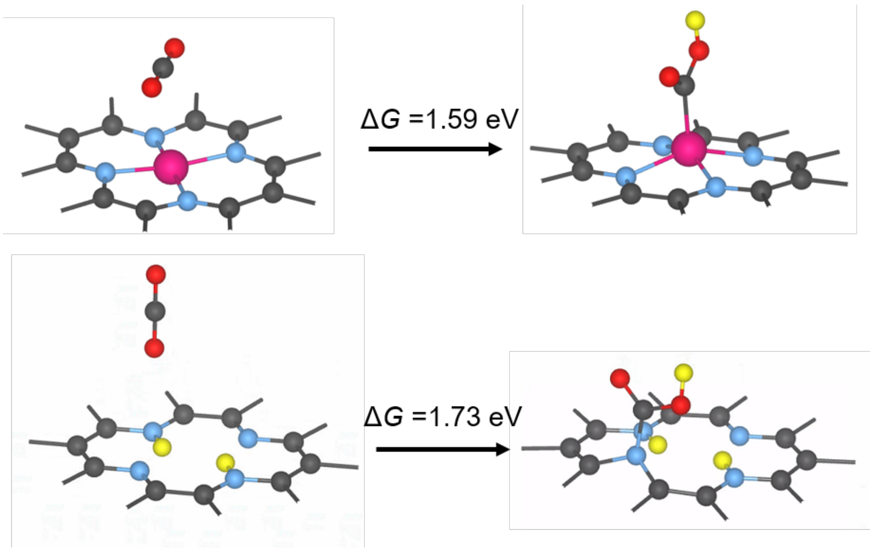

Supplementary Figure 36. The first hydrogenation step of CO_2RR on Cu_1/NC and NC. Color code: N, blue; C, black; H, yellow; O, red; Cu, pink.

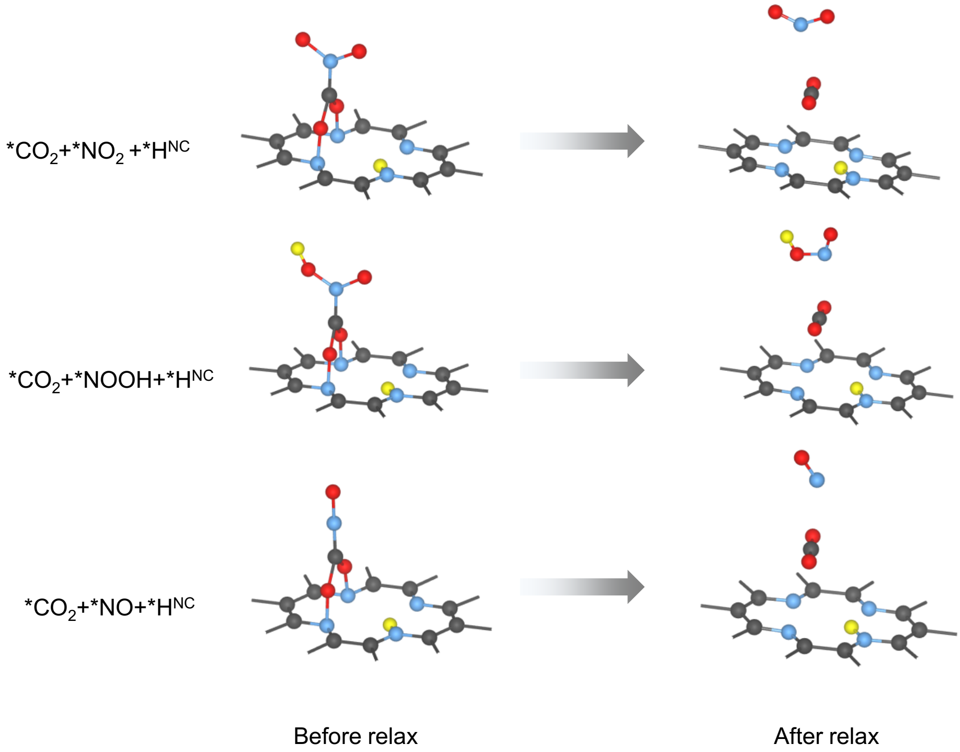

Supplementary Figure 37. C−N coupling between CO_2 and the main intermediates of NtrRR on NC. All the intermediates after the coupling are unstable. Color code: N, blue; C, black; H, yellow; O, red.

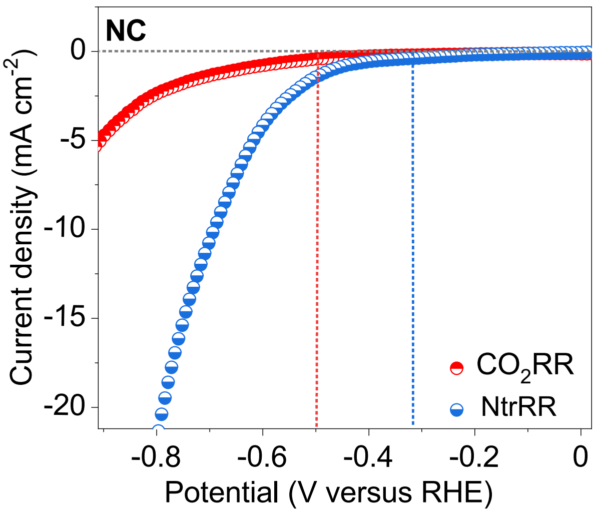

Supplementary Figure 38. LSV curves of individual NtrRR and individual CO_2RR on NC. The onset potentials for NtrRR and CO_2RR are estimated to be −0.32 and −0.50 V, respectively.

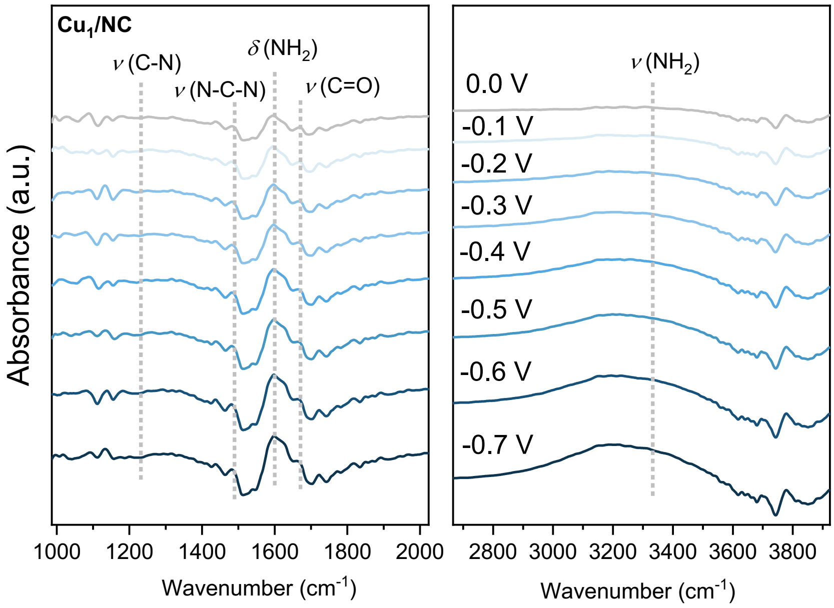

Supplementary Figure 39. ATR-SEIRAS spectra for Cu_1/NC under different applied potentials during co-reduction of CO_2 and NO_3^-.

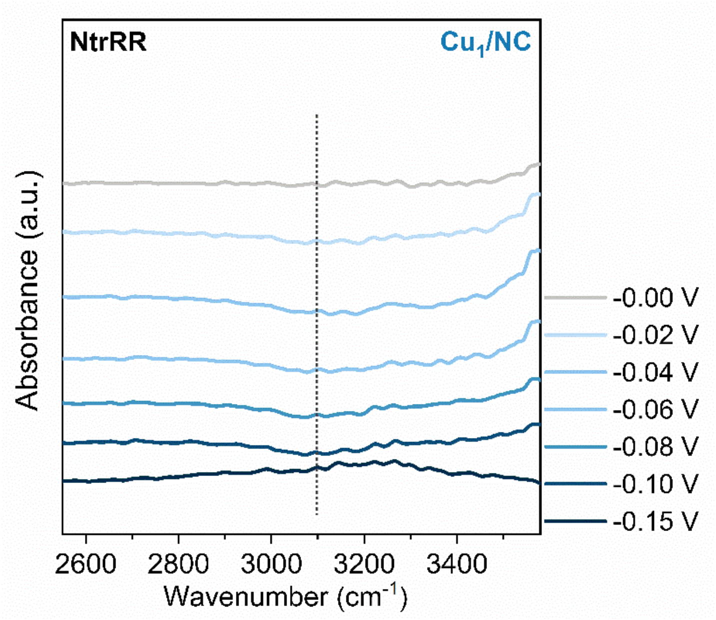

Supplementary Figure 40. ATR-SEIRAS spectra for Cu_1/NC in the range under relatively low applied potentials during individual NtrRR.

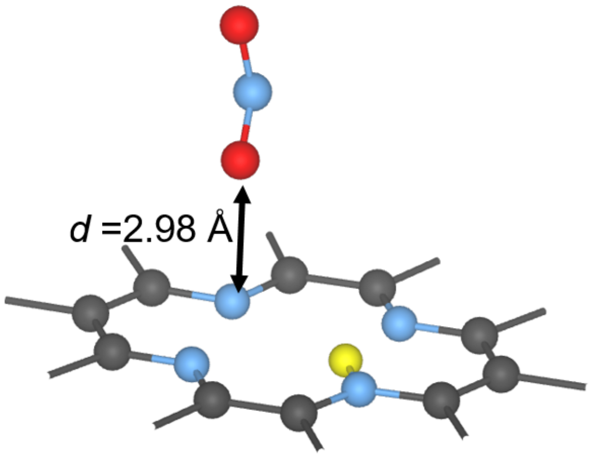

Supplementary Figure 41. The distance between *NO_2 and the NC slab. Color code: N, blue; C, black; H, yellow; O, red.

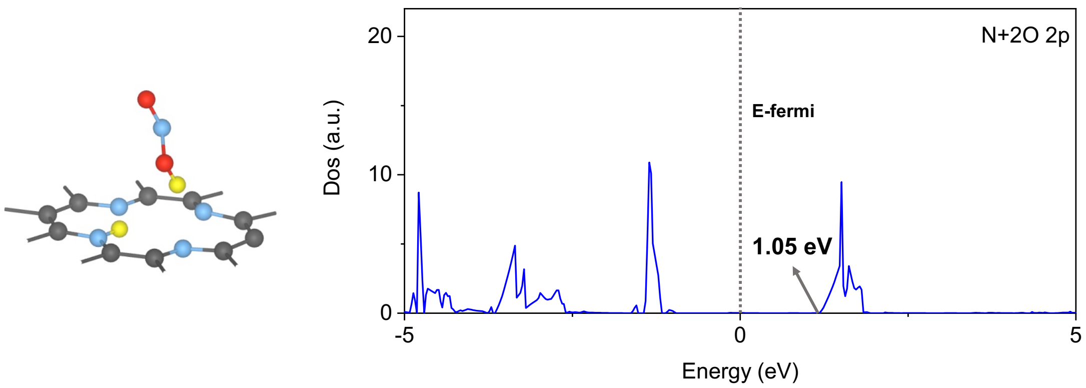

Supplementary Figure 42. The density of states (DOS) of the *NOOH configuration. Color code: N, blue; C, black; H, yellow; O, red.

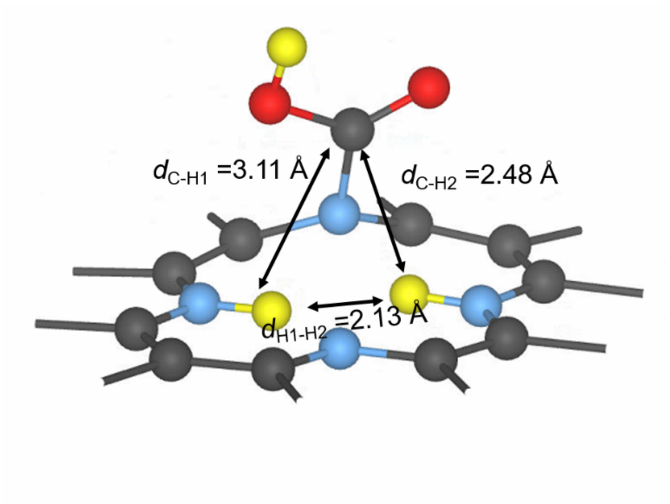

Supplementary Figure 43. The configuration of *COOH on N_4 site with two H^{NC} atoms. Color code: N, blue; C, black; H, yellow; O, red.

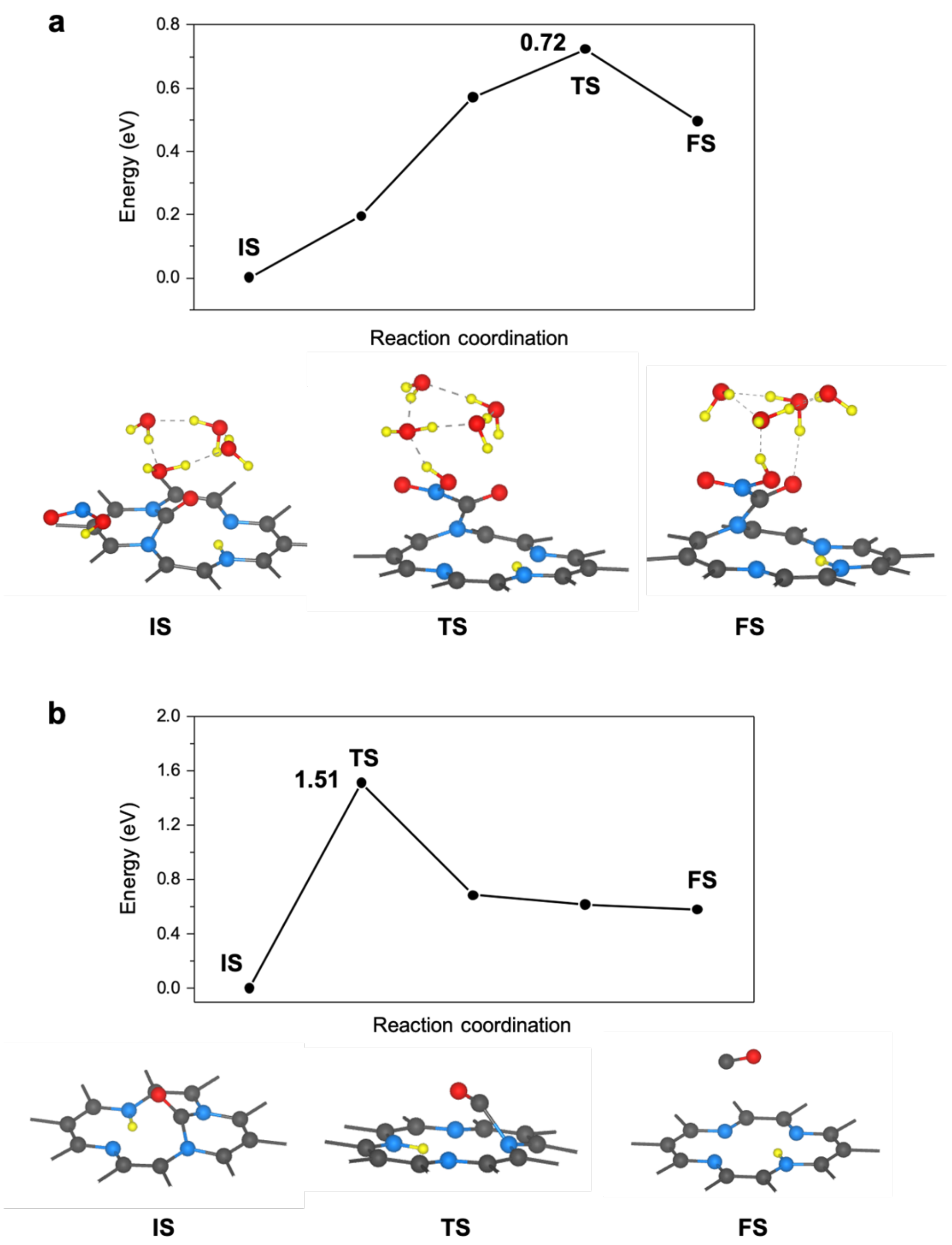

Supplementary Figure 44. CI-NEB calculations of (a) the first C−N coupling process and (b) *CO desorption process on NC. The structural configurations of the initial state (IS), transition state (TS) and final state (FS) are displayed. Color code: N, blue; C, black; H, yellow; O, red.

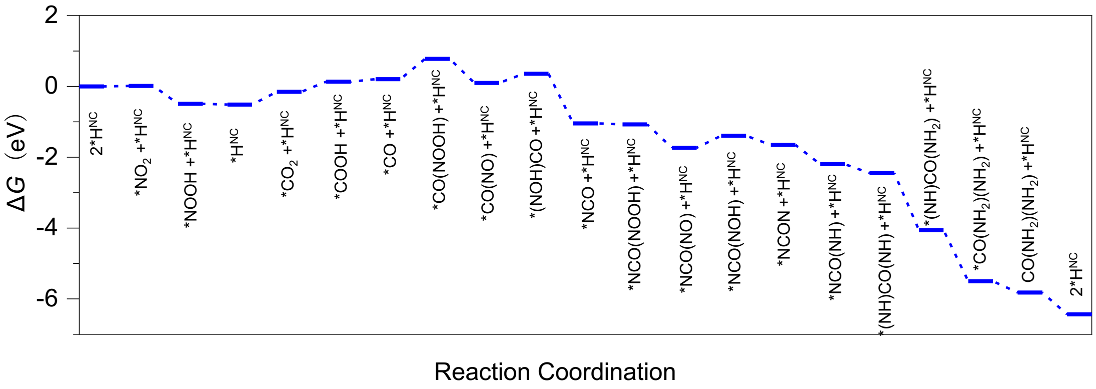

Supplementary Figure 45. The free energy diagram of the urea formation process on NC at 0 V versus RHE.

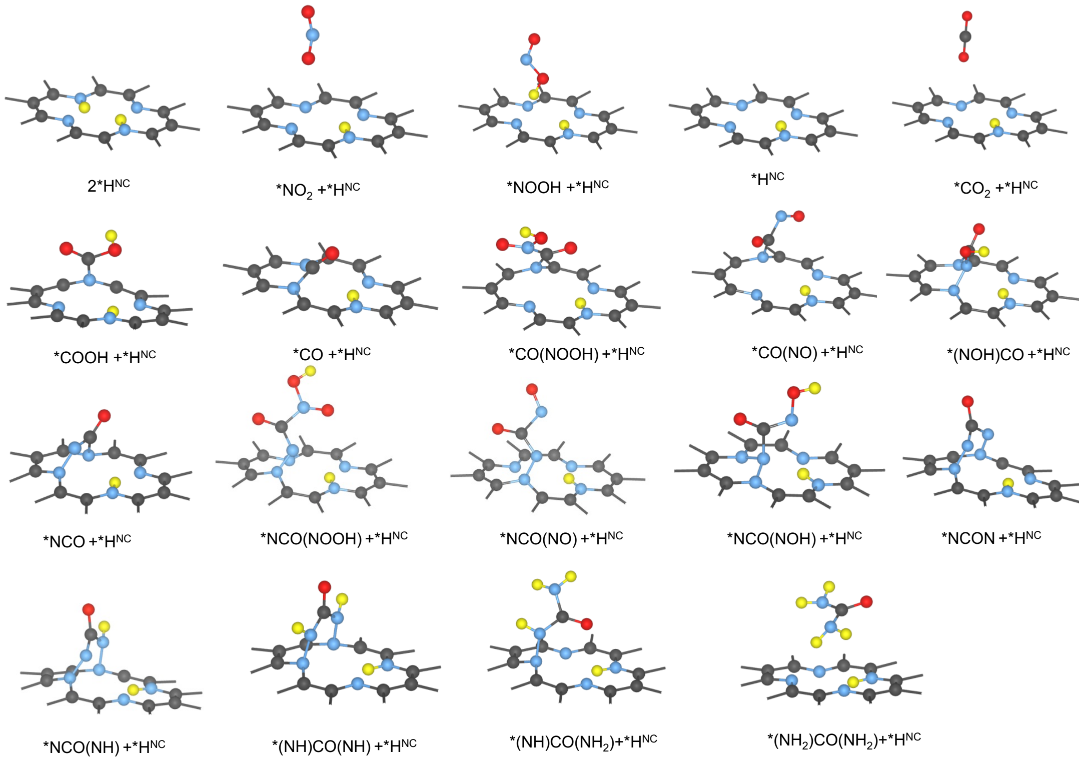

Supplementary Figure 46. The optimized atomic structures of the intermediates during urea formation on NC. Color code: N, blue; C, black; H, yellow; O, red.

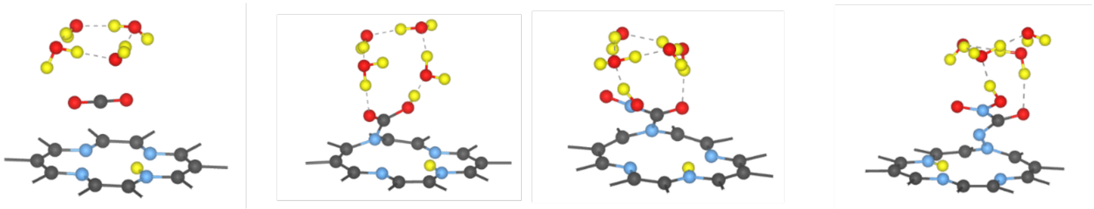

Supplementary Figure 47. The geometry of $*CO_2$, *COOH, *CONOOH and *CONNOOH with explicit surface water on NC. The dash line indicates the hydrogen bond. Color code: N, blue; C, black; H, yellow; O, red.

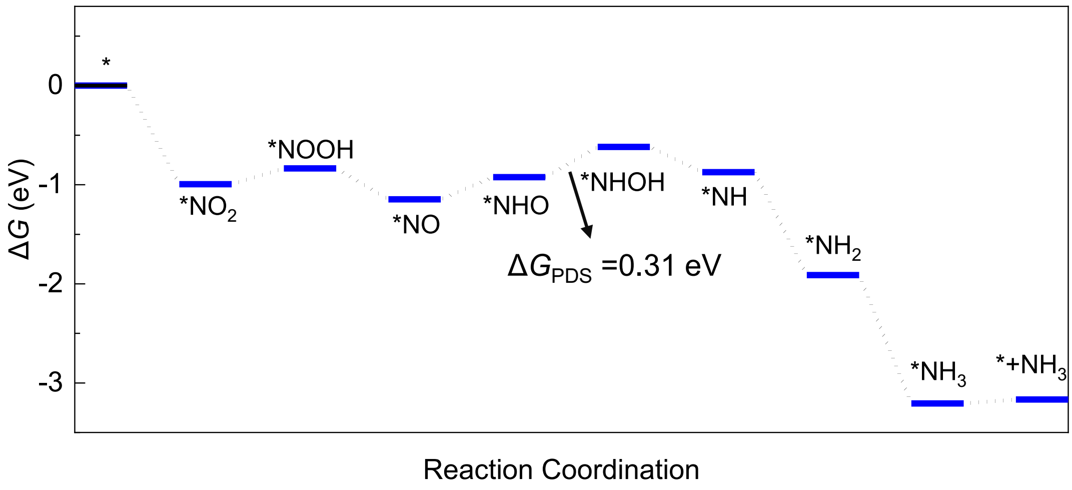

Supplementary Figure 48. The free energy diagram of NtrRR on Cu_1/NC at 0 V versus RHE.

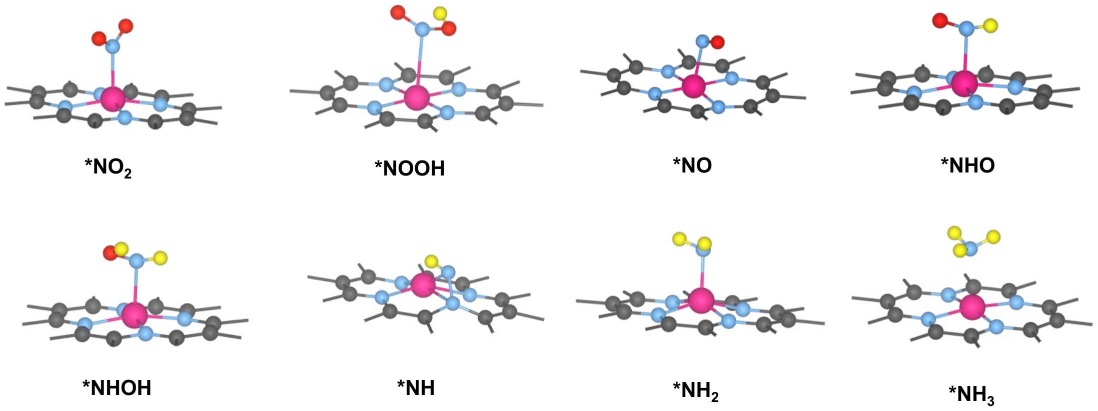

Supplementary Figure 49. The optimized atomic structures of intermediates during NtrRR on Cu_1/NC. Color code: N, blue; C, black; H, yellow; O, red; Cu, pink.

Supplementary Table 1. Comparison of electrochemical urea synthesis on different catalysts.

Catalyst	Reactant	Electrolyte	Yield rate	FE (%)	Ref.
NC	$NO_3^- + CO_2$	0.1 M $KHCO_3$ + 0.1 M KNO_3	596.1 μg $h^{-1}mg^{-1}$	62	This work
Cu_1/NC	$NO_3^- + CO_2$	0.1 M $KHCO_3$ + 0.1 M KNO_3	210 μg h^{-1} mg^{-1}	15.6	
Cu-GS-800	$NO_3^- + CO_2$	0.1 M $KHCO_3$ + 0.1 M KNO_3	1800 μg h^{-1} mg^{-1}_{cat}	28	7
Cu@Zn	$NO_3^- + CO_2$	0.2 M $KHCO_3$ + 0.1 M KNO_3	7.29 μmol h^{-1} cm^{-2}	9.28	8
AuPd	$NO_3^- + CO_2$	0.075 M $KHCO_3$ and 0.025 M KNO_3	204.2 μg h^{-1} mg^{-1}	15.6	9
Fe, Fe_3O_4/CNT	$NO_3^- + CO_2$	0.1 M KNO_3	1341.3 ± 112.6 μg h^{-1} mg_{cat}^{-1}	16.5±6.1	10
F-CNT	$NO_3^- + CO_2$	0.1 M KNO_3	381.6 μg h^{-1} mg_{cat}^{-1}	18	11
V_O-InOOH	$NO_3^- + CO_2$	0.1 M KNO_3	592.5 μg h^{-1} mg^{-1}	51	12
$In(OH)_3$	$NO_3^- + CO_2$	0.1 M KNO_3	533.1 μg h^{-1} mg^{-1}	54.3	13
CoPc-COF	$NO_3^- + CO_2$	0.3 M $KHCO_3$ 0.2 M KNO_3	753.1 μg h^{-1} mg^{-1}	49	14
$Cu_{97}In_3$	$NO_3^- + CO_2$	0.1 M $KHCO_3$ 10 mM KNO_3	786 μg h^{-1} mg^{-1}	<10	15
In_2O_3/Graphene	$NO_3^- + CO_2$	0.1 M $KHCO_3$ + 0.1 M KNO_3	357.5μg h^{-1} mg^{-1}	10.46	16
Te–Pd NCs	$NO_2^- + CO_2$	0.1 M $KHCO_3$ + 0.01 M KNO_2	N/A	12.2%	17
Cu-TiO_2	$NO_2^- + CO_2$	0.2 M $KHCO_3$ + 0.02 M KNO_2	20.8 μmol · h^{-1}	43.1	18
AuCu SANFs	$NO_2^- + CO_2$	0.01 M KNO_2	3889.6 μg h^{-1} mg_{cat}^{-1}	24.7	19
Zn/GDL	NO + CO_2	0.2 M $KHCO_3$	907.8 μg h^{-1} mg^{-1}	11.26	20
Pd_1Cu_1/TiO_2	$N_2 + CO_2$	0.1 M $KHCO_3$	201.6 μg h^{-1} mg^{-1}	8.92	21
Bi/$BiVO_4$	$N_2 + CO_2$	0.1 M $KHCO_3$	354.6 μg h^{-1} mg^{-1}	12.55	22
$BiFeO_3/BiVO_4$	$N_2 + CO_2$	0.1 M $KHCO_3$	296.4 μg h^{-1} mg^{-1}	17.18	23
CuPc NTs	$N_2 + CO_2$	0.1 M $KHCO_3$	143.47μg h^{-1} mg^{-1}	12.99	24
InOOH	$N_2 + CO_2$	0.1 M $KHCO_3$	411.0 μg h^{-1} mg^{-1}	20.97	25
$Ni_3(BO_3)_2$	$N_2 + CO_2$	0.1 M $KHCO_3$	582.0 μg h^{-1} mg^{-1}	20.36	26

Supplementary references

1 Lv, C. *et al.* Defect engineering metal-free polymeric carbon nitride electrocatalyst for effective nitrogen fixation under ambient conditions. *Angew. Chem. Int. Ed.* **130**, 10403-10407 (2018).

2 Wang, C. *et al.* One-pot synthesis of N-doped graphene for metal-free advanced oxidation processes. *Carbon* **102**, 279-287 (2016).

3 Rahmatullah, M. & Boyde, T. Improvements in the determination of urea using diacetyl monoxime; methods with and without deproteinisation. *Clin. Chim. Acta* **107**, 3-9 (1980).

4 Ivančič, I. & Degobbis, D. An optimal manual procedure for ammonia analysis in natural waters by the indophenol blue method. *Water Res.* **18**, 1143-1147 (1984).

5 Chen, S. *et al.* Electrocatalytic synthesis of ammonia at room temperature and atmospheric pressure from water and nitrogen on a carbon-nanotube-based electrocatalyst. *Angew. Chem. Int. Ed.* **129**, 2743-2747 (2017).

6 Watt, G. W. & Chrisp, J. D. Spectrophotometric method for determination of hydrazine. *Anal. Chem.* **24**, 2006-2008 (1952).

7 Leverett, J. *et al.* Tuning the coordination structure of Cu-N-C single atom catalysts for simultaneous electrochemical reduction of CO_2 and NO_3^- to urea. *Adv. Energy Mater.* **12**, 2201500 (2022).

8 Meng, N. *et al.* Oxide-derived core–shell Cu@Zn nanowires for urea electrosynthesis from carbon dioxide and nitrate in water. *ACS Nano* **16**, 9095-9104 (2022).

9 Wang, H. *et al.* Realizing efficient CN coupling via electrochemical co-reduction of CO_2 and NO_3^- on AuPd nanoalloy to form urea: Key CN coupling intermediates. *Appl. Catal. B* **318**, 121819 (2022).

10 Geng, J. *et al.* Ambient electrosynthesis of urea with nitrate and carbon dioxide over iron-based dual-sites. *Angew. Chem. Int. Ed.* **62**, e202210958 (2022).

11 Liu, X. *et al.* Carbon nanotubes with fluorine-rich surface as metal-free electrocatalyst for effective synthesis of urea from nitrate and CO_2. *Appl. Catal. B* **316**, 121618 (2022).

12 Lv, C. *et al.* A defect engineered electrocatalyst that promotes high-efficiency urea synthesis under ambient conditions. *ACS Nano* **16**, 8213-8222 (2022).

13 Lv, C. *et al.* Selective electrocatalytic synthesis of urea with nitrate and carbon dioxide. *Nat. Sustain.* **4**, 868-876 (2021).

14 Li, N. *et al.* Metalphthalocyanine frameworks grown on TiO_2 nanotubes for synergistically and efficiently electrocatalyzing urea production from CO_2 and nitrate. *Sci. China Chem.* **66**, 1417-1424 (2023).

15 Liu, Y. *et al.* C-Bound or O-Bound Surface: Which One Boosts Electrocatalytic Urea Synthesis? *Angew. Chem. Int. Ed.* **62**, e202300387 (2023).

16 Mao, Y. *et al.* Ambient electrocatalytic synthesis of urea by co-reduction of NO_3^- and CO_2 over graphene-supported In_2O_3. *Chinese Chem. Lett.* 108540 (2023).

17 Feng, Y. *et al.* Te-doped Pd nanocrystal for electrochemical urea production by efficiently coupling carbon dioxide reduction with nitrite reduction. *Nano Lett.* **20**, 8282-8289 (2020).

18 Cao, N. *et al.* Oxygen vacancies enhanced cooperative electrocatalytic reduction of carbon dioxide and nitrite ions to urea. *J. Colloid Interface Sci.* **577**, 109-114 (2020).

19 Liu, S. *et al.* AuCu nanofibers for electrosynthesis of urea from carbon dioxide and nitrite. *Cell Rep. Phys. Sci.* **3**, 100869 (2022).

20 Huang, Y. *et al.* Direct electrosynthesis of urea from carbon dioxide and nitric oxide. *ACS Energy Lett.* **7**, 284-291 (2021).

21 Chen, C. *et al.* Coupling N_2 and CO_2 in H_2O to synthesize urea under ambient conditions. *Nat. Chem.* **12**, 717-724 (2020).

22 Yuan, M. *et al.* Unveiling electrochemical urea synthesis by co-activation of CO_2 and N_2 with Mott–Schottky heterostructure catalysts. *Angew. Chem. Int. Ed.* **133**, 11005-11013 (2021).

23 Yuan, M. *et al.* Electrochemical C–N coupling with perovskite hybrids toward efficient urea synthesis. *Chem. Sci.* **12**, 6048-6058 (2021).

24 Ghorai, U. *et al.* Electrosynthesis of green urea by co-reduction of N_2 and CO_2 using dual active sites of copper phthalocyanine nanotube. *ChemRxiv* (2021).

25 Yuan, M. *et al.* Artificial frustrated Lewis pairs facilitating the electrochemical N_2 and CO_2 conversion to urea. *Chem Catal.* **2**, 309-320 (2022).

26 Yuan, M. *et al.* Highly selective electroreduction of N_2 and CO_2 to urea over artificial frustrated Lewis pairs. *Energy Environ. Sci.* **14**, 6605-6615 (2021).
